# How reactive is water at the nanoscale and how to control it?

**DOI:** 10.1126/sciadv.aeb5772

**Published:** 2026-06-24

**Authors:** Xavier R. Advincula, Yair Litman, Kara D. Fong, William C. Witt, Christoph Schran, Angelos Michaelides

**Affiliations:** ^1^ Yusuf Hamied Department of Chemistry, University of Cambridge, Lensfield Road, Cambridge CB2 1EW, UK.; ^2^ Cavendish Laboratory, Department of Physics, University of Cambridge, Cambridge CB3 0HE, UK.; ^3^ Lennard-Jones Centre, University of Cambridge, Trinity Lane, Cambridge CB2 1TN, UK.; ^4^ Max Planck Institute for Polymer Research, Ackermannweg 10, 55128 Mainz, Germany.; ^5^ Division of Chemistry and Chemical Engineering, California Institute of Technology, Pasadena, CA 91125, USA.; ^6^ Harvard John A. Paulson School of Engineering and Applied Sciences, Harvard University, Cambridge, MA, USA.

## Abstract

Nanoconfined water plays a key role in nanofluidics, electrochemistry, and catalysis, yet its reactivity remains a matter of debate. Prior studies have reported both enhanced and suppressed water self-dissociation relative to the bulk, but without a consistent explanation. Here, using enhanced sampling molecular dynamics with machine-learned potentials trained at first-principles accuracy, we investigate dissociation behavior in water confined within two-dimensional slit pores and nanodroplets, using graphene and hexagonal boron nitride as model materials. We find that reactivity is extremely sensitive to water density, geometry, and surface chemistry, among other factors. Despite this complexity, we show that chemical potential, together with interfacial interactions, governs dissociation trends and explains the variability observed in prior studies. Within this framework, when confined water is compared to the bulk at equivalent chemical potential, corresponding to thermodynamic equilibrium with a bulk reservoir, its reactivity remains essentially unchanged; rather, differences arise when the systems are compared at different chemical potentials or under distinct interfacial conditions. This thermodynamic perspective reconciles previous contradictions and reveals how nanoscale environments can drastically shift water reactivity. Our findings provide molecular-level insight and offer a design lever for modulating water chemistry at the nanoscale.

## Teaser

Water reactivity at the nanoscale shows that the devil is in the details, as subtle changes can radically influence its behavior.

## INTRODUCTION

Water is central to chemical and biological function, not only as a medium but also as an active participant in countless processes. Among its most fundamental properties is the spontaneous self-dissociation into hydronium (H_3_O^+^) and hydroxide (OH^−^) ions, a reaction that defines pH and drives acid-base chemistry, proton transport, and catalytic behavior across a vast range of systems (*1*–*4*). The equilibrium constant for this process, *K*_w_, governs the balance between neutral and ionized species in aqueous environments. Although well-characterized in bulk water (*1*, *5*–*9*), many other real-world environments, such as biological membranes, mineral interfaces, nanopores, and catalytic surfaces, feature water confined to nanometer-scale dimensions, where water’s self-dissociation is much less well explored.

At the nanoscale, confinement can drastically influence water’s structural (*10*–*12*), dynamical (*13*–*16*), and dielectric properties (*17*, *18*), often leading to behavior that departs markedly from that of bulk water. These effects arise across a wide range of systems that vary in the nature and rigidity of their confining environments. For example, in soft environments such as biomolecular cavities or atmospheric aerosols, interfaces are typically flexible and chemically heterogeneous. By contrast, rigid confinements like carbon nanotubes and two-dimensional (2D) slit pores impose well-defined geometric constraints. The nature of confinement has a direct impact on solvation structure, interfacial interactions, and ultimately, chemical reactivity (*19*, *20*). While reactivity in soft environments has been widely explored (*19*, *21*–*23*), much less is known about fundamental proton-transfer processes such as water self-dissociation in rigid confinement. In rigid media, geometric constraints can disrupt the hydrogen-bond network, alter solvation, and shift the equilibrium between neutral and dissociated species (*20*, *24*). These effects offer the possibility of modulating water reactivity through confinement alone. This behavior is especially relevant in nanofluidic channels (*25*), electrochemical systems (*26*–*28*), and surface catalysis on 2D materials (*3*, *29*). Even modest deviations from bulk-like behavior in these settings can lead to emergent reactivity patterns (*20*), highlighting the need to understand water’s behavior under nanoscale confinement for the design of functional interfaces and reaction environments.

**Fig. 1.**
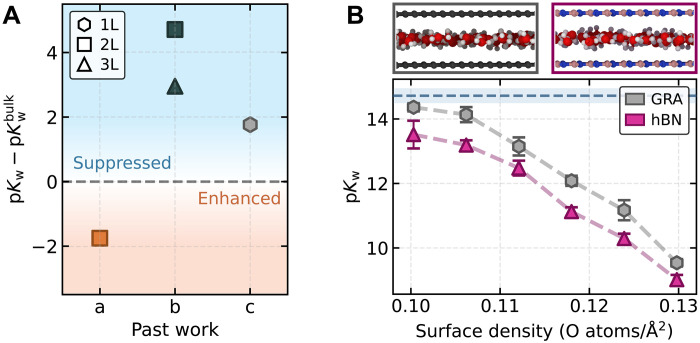
The reactivity of nanoconfined water depends sensitively on the nature of the confinement. (**A**) Reported p*K*_w_ values for nanoconfined water from prior studies, illustrating both suppression and enhancement of self-dissociation relative to bulk water. References: (a) Muñoz-Santiburcio and Marx (*32*), (b) Di Pino *et al.* (*33*), and (c) Dasgupta *et al.* (*34*) These studies involve different confining environments: a corresponds to layered minerals, whereas b and c examine graphene-based systems using QM/MM and MLP approaches, respectively. 1L, 2L, and 3L indicate the number of confined water layers formed within the slit pores, corresponding to monolayer, bilayer, and trilayer water, respectively. (**B**) Dependence of p*K*_w_ on the surface density for monolayer water confined in rigid graphene (GRA) and hBN slit pores, highlighting the influence of both material type and density. The horizontal dashed blue line indicates our bulk reference estimate at 1 bar with its error.

Although it is established that confinement can substantially alter the structural and dynamical properties of water, its influence on self-dissociation remains an open question. First-principles molecular dynamics (MD) simulations have provided important insights, particularly in geometrically well-defined environments such as carbon nanotubes (*30*, *31*) and 2D slit pores composed of chemically inert materials such as mackinawite or graphene (*32*–*34*). However, these studies have reported markedly different outcomes. Muñoz-Santiburcio and Marx (*32*) found a substantial, 55-fold enhancement in the rate of water self-dissociation when confined as a bilayer between mackinawite sheets, attributing this acceleration directly to nanoconfinement. This corresponds to a pronounced decrease in p*K*_w_, which reflects a greater degree of ionization in water. In contrast, Di Pino *et al.* (*33*) reported no enhancement, and even suppression, when water was confined between graphene layers, arguing that earlier results may have been influenced by overpressurization rather than confinement per se. More recently, Dasgupta *et al.* (*34*) arrived at a similar conclusion, showing that water confined to a subnanometer monolayer exhibits strongly reduced dissociation. This corresponds to an increase in p*K*_w_, indicating lower concentrations of dissociated ionic species under such extreme confinement. These contrasting findings, illustrated in Fig. 1A, emphasize the complexity of confined aqueous systems, where dissociation behavior arises from a subtle interplay of environmental and thermodynamic factors. Disentangling these contributions and isolating the specific role of confinement remains a central challenge. Adding to this uncertainty, indirect experimental studies have also yielded contrasting interpretations (*18*, *35*, *36*). Because of the indirect nature of these measurements and their sensitivity to local conditions, these findings remain difficult to reconcile, contributing to a fragmented picture of how nanoconfinement influences water reactivity. Together, these discrepancies highlight the need for a consistent and well-controlled framework to determine how confinement affects water self-dissociation: one capable of capturing both molecular-scale structure and thermodynamic factors that govern dissociation equilibria across a range of conditions.

In response to these challenges, we investigate the key factors that govern the self-dissociation of water under nanoconfinement. To navigate this complex landscape, we use carefully developed and validated machine learning potentials (MLPs), trained on density functional theory reference data (see Materials and Methods), which enable large-scale MD simulations with near first-principles accuracy. We focus on two archetypal materials for studying nanoconfined water: graphene and hexagonal boron nitride (hBN), as their similar interface structures contrast with the different behavior that water exhibits near their surfaces (*37*–*39*). We first examine the key factors that complicate direct comparisons between confined and bulk water, including variations in density, geometry, and confining material, among others. From this extensive set of simulations, a strong sensitivity of water to the nature of the confinement is highlighted. By analyzing dissociation behavior as a function of chemical potential in monolayer graphene slit pores, we show that confinement alone does not inherently alter water’s acid-base chemistry. Instead, we find that, when confined water is compared to the bulk at equivalent chemical potential, corresponding to thermodynamic equilibrium with a bulk reservoir, it exhibits dissociation behavior that closely resembles that of the bulk. We then extend our study to more complex geometries, including material-encapsulated nanodroplets, which introduce interfacial curvature, spatial heterogeneity, and edge reactivity, and represent another class of experimentally realizable nanoconfinement systems. In these droplets, we find that variations in surface chemistry and local structure can lead to markedly distinct departures from bulk-like behavior. Notably, at hBN interfaces, we identify an alternative dissociative pathway in which hydroxide ions produced during self-dissociation are stabilized through chemisorption at droplet edges. This finding highlights that interfacial chemistry, rather than geometric confinement alone, offers a powerful means of tuning water reactivity at the nanoscale. Building on these insights, we illustrate how interfacial geometry, chemistry, and local structure influence dissociation equilibria in confined systems. Together, our findings reveal when confined water behaves like the bulk and when it departs from it, offering molecular-scale principles for understanding and controlling acid-base behavior under confinement.

## RESULTS

### Strong sensitivity of nanoconfined water to the confinement conditions

The question “How reactive is water at the nanoscale?” is not a simple one to answer because many factors can, in principle, influence the tendency of water to dissociate. To tackle this question, we started by systematically exploring slit pore geometries with water at different densities and in different confining materials (graphene and hBN). Since water self-dissociation is a rare event on accessible simulation timescales, we use umbrella sampling to enhance sampling along a reaction coordinate associated with the dissociation process. Specifically, we bias the coordination number of a water oxygen, *n*_H_, which tracks the number of hydrogen atoms covalently bonded to it (*40*). This variable captures the transition from a neutral H_2_O molecule to the ionized species H_3_O^+^ and OH^−^. By sampling along *n*_H_, we reconstruct the dissociation pathway and compute the corresponding free energy profiles. From these, we extract the dissociation constant, p*K*_w_, using the relation $pK_{w}=\Delta F^{‡}/\left(\mathrm{RT}\text{ln}10\right)$, where Δ*F*^‡^ is the free energy barrier between reactant and product states (*8*, *41*) (see Materials and Methods for details). Our computational approach shows excellent agreement with experiment for the bulk dissociation constant as a function of temperature, as discussed in detail in section S2.

The results of our systematic analysis of dissociation constants are summarized in Fig. 1. Let us start by considering how density influences water self-dissociation of monolayer-confined water in rigid graphene slit pores (see section S1 for setup details). Density is difficult to define in nanoconfined systems, and consequently, we explored a range of water densities across our simulations. Even in experiments, a range of densities can be expected depending on the conditions used to create the systems of interest. To facilitate an initial comparison of trends, we considered bulk-like density variations ranging from ~0.9 to 1.2 g/cm^3^. However, in our analysis, we focus on variations in surface density, defined as the total number of oxygen atoms (i.e., water molecules) in the slit divided by the lateral area of the confining surfaces (see section S1). This quantity is well defined under confinement and avoids the ambiguities associated with estimating an effective liquid volume, which would require assigning an arbitrary thickness to the confined water layer to define a volumetric density (*42*). Using this definition, Fig. 1B shows that increasing the surface density leads to a systematic decrease in the p*K*_w_. At a fixed confinement width, increasing the surface density effectively compresses the confined water layer, resulting in higher effective pressures within the slit. Thus, this trend suggests that pressure plays a key role in enhancing self-dissociation under confinement and mirrors the known behavior of bulk water, where elevated pressure lowers p*K*_w_ and promotes dissociation (*43*). However, as we will explore in more detail later, drawing such parallels requires a careful comparison.

To examine the influence of the confining material, we also consider hBN slit pores. The same qualitative trend is observed: Higher density leads to a lower p*K*_w_. However, consistently lower p*K*_w_ values are found compared to graphene. This difference highlights the role of surface chemistry, such as hydrophobicity or hydrogen-bonding characteristics, in shaping the local reactivity environment. We have also extended this analysis to different pore widths, comparing monolayer (1L), bilayer (2L), and trilayer (3L) water confined in rigid graphene slit pores (see section S4). Because selecting a unique equilibrium water content across different pore widths is inherently nontrivial, these systems are constructed at fixed 2D surface density, with the bilayer and trilayer pores containing approximately two and three times the monolayer loading, respectively. These results highlight that dissociation is also sensitive to confinement width and water loading. However, the detailed trends depend strongly on the underlying thermodynamic conditions, consistent with a recent systematic study of the effect of water filling on dissociation in slit pores (*44*).

Overall, these findings reveal the intricate interplay of density, pressure, surface chemistry, confinement geometry, and phase behavior. This complexity not only makes direct comparisons with bulk water challenging but also helps explain the conflicting results reported in the literature, highlighting the importance of evaluating confined systems under thermodynamically consistent conditions.

### Confined and bulk water show similar dissociation behavior

**Fig. 2.**
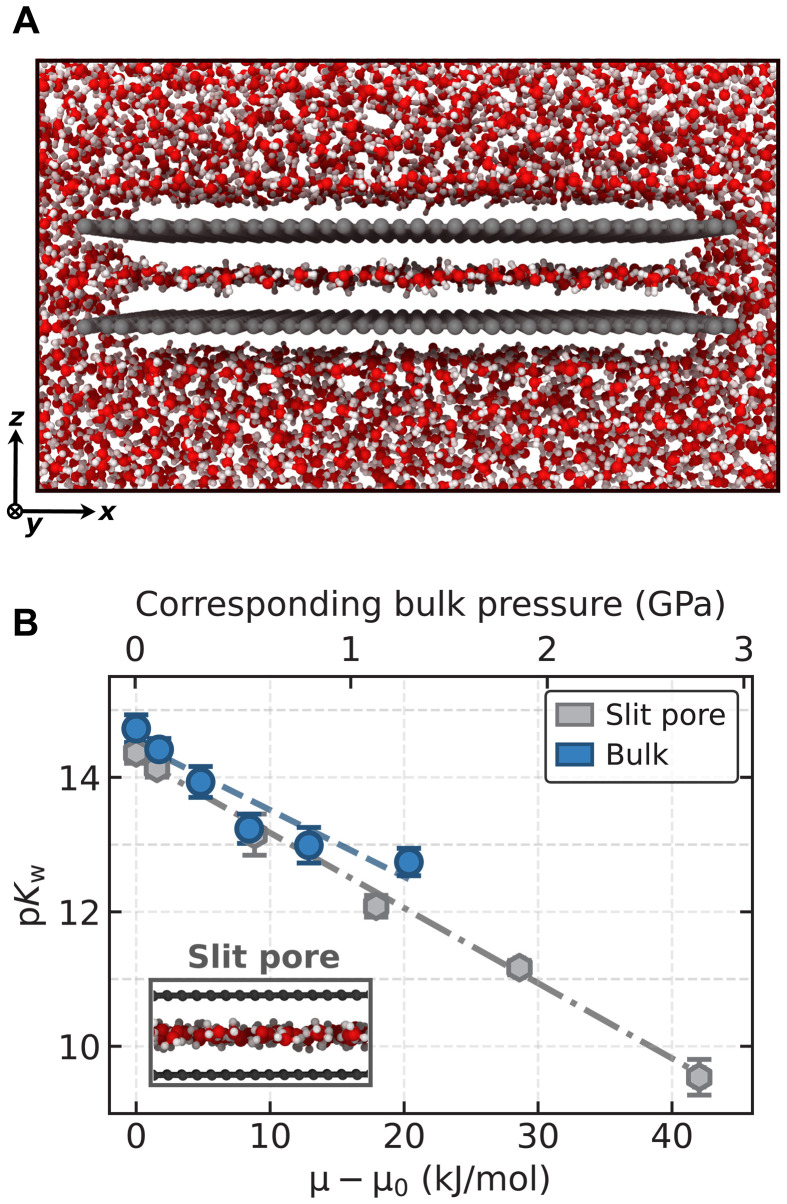
Comparison of water self-dissociation in bulk and nanoconfined environments under equivalent thermodynamic conditions. (**A**) Representative simulation snapshot showing the type of system used to equilibrate confined water: a rigid graphene slit pore with armchair edges immersed in a bulk water reservoir in the NPT ensemble (see sections S1 and S5 for further details). This approach enables consistent determination of a chemical potential reference for the confined system, allowing direct comparison with bulk water (see Materials and Methods). (**B**) p*K*_w_ as a function of the chemical potential difference relative to the corresponding reference, shown for both bulk water and graphene slit pores. The accompanying simulation snapshot shows the graphene slit pore systems simulated for comparison with bulk water, consisting of monolayer water confined between rigid, parallel graphene sheets (see section S1). The corresponding bulk pressure is indicated on the secondary (top) axis. The dashed lines represent linear fits to guide the eye.

Because water self-dissociation is an equilibrium process, meaningful comparisons between bulk and confined environments require a consistent thermodynamic basis. To this end, we focus on the chemical potential, μ, which governs the equilibrium distribution of molecular and ionic species and offers a natural reference point for comparing dissociation behavior across different conditions (*45*). Rather than attempting to match pressure in nanoconfined systems, we analyze how p*K*_w_ varies relative to a reference chemical potential, μ – μ_0_. Pressure-based comparisons in confined geometries are challenging because defining an effective volume or slit width is inherently ambiguous, a difficulty that is also well known in the context of defining dielectric response under nanoconfinement (*46*, *47*). Accordingly, we focus specifically on monolayer water confined between rigid graphene sheets, which offers a controlled environment for isolating the thermodynamic factors that influence dissociation. For bulk water, μ_0_ corresponds to the chemical potential of liquid water at 1 bar, a well-established reference in both experiments and simulations. For confined water, μ_0_ corresponds to the chemical potential at which the surface density in the central region of the slit pore matches its equilibrium value when the system is in contact with a bulk water reservoir at 1 bar, thereby placing the confined system in thermodynamic equilibrium with the bulk. This ensures that both systems are compared at equivalent chemical potential. To determine the equilibrium density in confined water, we simulate a series of systems consisting of two parallel graphene layers, which are periodic along the *y* axis and immersed in a bulk water reservoir, using the NPT ensemble with pressure maintained at 1 bar (Fig. 2A). Because of volume fluctuations inherent to the NPT ensemble, the total simulation box dimensions vary, reaching up to ~105.742 Å × 77.004 Å × 36.000 Å (≈30,000 atoms). This highlights the key role of MLPs in this work, especially recent advances in large-scale models used here (see Materials and Methods), in enabling simulations at this scale. By computing the surface density at the center of the slit and extrapolating to the infinite-size limit, we obtain a thermodynamically consistent reference state for the confined system.

With these reference points established, we systematically vary the chemical potential away from μ_0_ in both bulk and confined setups and compute the corresponding p*K*_w_ values (see section S5 for details). For the confined cases, we focus on monolayer water situated between rigid, parallel graphene sheets in a periodic slit pore geometry (similar to the setups introduced in Fig. 1; see section S1). This controlled setup enables a direct comparison of dissociation behavior across environments under matched thermodynamic conditions, avoiding the ambiguities associated with pressure- or density-based comparisons. As shown in Fig. 2B, once framed in terms of μ – μ_0_, the dissociation behavior of confined water is similar to that of the bulk. This finding indicates that confinement alone does not inherently enhance or suppress water self-dissociation. Rather, the differences observed across environments primarily reflect shifts in the underlying thermodynamic state imposed by confinement.

### Controlling dissociation equilibria in experimentally realizable systems

Having established a thermodynamically consistent framework to interpret water dissociation across bulk and confined systems, we now pose a broader question: Can this understanding be used to modulate reactivity at the nanoscale? To explore this, we complement our analysis of slit pores by investigating material-encapsulated nanodroplets, a distinct form of nanoconfinement that introduces interfacial curvature, van der Waals (vdW) pressure, and spatial heterogeneity. These characteristics provide additional degrees of control over the local environment and offer a useful comparison to planar confinement. In addition to their relevance as experimentally realizable systems (e.g., nanocapillaries) (*10*), nanodroplets also serve as representative models for intercalated water layers found in nanofluidic and nanoelectronic devices, as well as in energy storage systems such as batteries and supercapacitors, where confined water plays a key role in mediating wetting, transport, and chemical reactivity (*48*–*51*).

**Fig. 3.**
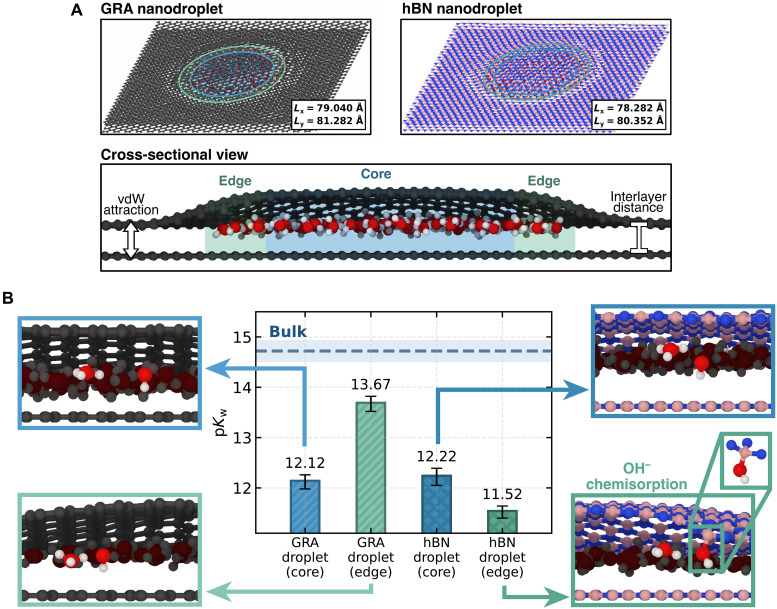
Control of water dissociation in nanodroplets. (**A**) Representative snapshots of water confined within graphene and hBN nanodroplets. The full system and cross-sectional views illustrate the droplet geometry, including lateral dimensions (*L_x_* and *L_y_*). These nanodroplets represent a distinct and experimentally realizable form of confinement, complementary to slit pores. Core and edge regions of the droplets are indicated by their respective color coding. (**B**) Comparison of the p*K*_w_ for bulk water and for water confined in graphene and hBN nanodroplets. The horizontal dashed blue line marks our bulk reference estimate at 1 bar (corresponding to the equilibrium bulk density), along with its associated uncertainty. In the nanodroplet systems, dissociation is induced either at the core or edge to probe spatial variations in water self-dissociation (see section S7). Snapshots for each case are shown, highlighting that edge dissociation in hBN leads to OH^−^chemisorption on the surface.

Figure 3A shows representative configurations of the graphene and hBN nanodroplets studied in this work. Each system consists of a rigid lower substrate, representing a supported 2D material, and a flexible top sheet. In the absence of water, the vdW attraction brings the sheets into close contact. When water is present, however, the interlayer spacing expands locally to accommodate the fluid, forming a droplet-like structure. These setups are constructed to ensure that the density in the central region of the droplet converges with respect to droplet size, reaching a well-defined equilibrium density that can be meaningfully compared to the equilibrium density of bulk water (see section S6 for details).

We now compare the dissociation behavior in these nanodroplet systems to that of bulk water. Within each droplet, dissociation events can occur either in the core or near the edge, where the local solvation environments differ substantially. We begin our discussion with the droplet core. As shown in Fig. 3B, for both graphene and hBN systems, we observe a decrease in p*K*_w_ of roughly 2.5 units compared to bulk. Given the logarithmic scale of p*K*_w_, this reflects a substantial enhancement in water self-dissociation within the interior of the droplet. This comparison is made relative to bulk water under standard conditions and reflects the higher effective pressure imposed on the confined water by the nanodroplet geometry, as discussed in the next section. At the droplet edge, however, the behavior shifts. In graphene nanodroplets, moving from the core to the edge leads to a p*K*_w_ increase of ~1.5 units, indicating suppressed dissociation near the droplet periphery. A similar shift has been reported for water droplets exposed to air, where moving from the droplet interior to the interface results in an increase of ~1.5 p*K*_w_ units (*23*). In the confined nanodroplets examined here, this shift is associated with interfacial structural changes, namely, a reduction in the coordination number and a decrease in hydrogen-bond connectivity at the droplet edge, leading to the emergence of undercoordinated interfacial water molecules (see section S7 and figs. S16 and S17). Although the microscopic details differ between air-water and graphene-water interfaces (*52*, *53*), both systems illustrate that undercoordination and hydrogen-bond disruption at an interface can disfavor water self-dissociation. A similar solvation-driven increase in p*K*_w_ under subnanometer confinement near graphene has been reported previously (*33*). Such interfacial structural effects are expected to modify the local electrostatic environment experienced by water molecules and proton defects, thereby shifting the free energy balance of water autoionization.

In hBN nanodroplets, we observe a starkly contrasting trend: moving from the core to the edge leads to a decrease in p*K*_w_, indicating enhanced dissociation near the interface. This behavior arises from a distinct dissociative pathway in which the OH^−^ ion produced during water dissociation becomes covalently bound to a boron atom at the droplet edge via chemisorption (*39*, *54*), as shown in the simulation snapshot in Fig. 3B. This binding lowers the free energy of the dissociated state and reduces the likelihood of recombination, thereby shifting the dissociation equilibrium, as evidenced by the plateau in the free energy profile (fig. S14). This mechanism is not observed in the droplet core, where the hBN surface remains locally planar and chemically inert, leading to dissociation behavior similar to that of graphene. It occurs preferentially at the droplet edge, where local curvature and strain alter boron hybridization and enhance surface reactivity. These observations align with prior studies of hydroxide adsorption on hBN (*39*, *54*).

### Unifying dissociation thermodynamics and routes to modulation

Until now, our analysis has primarily focused on how interfacial conditions and surface chemistry influence water self-dissociation. While these factors clearly affect reactivity, the consistently reduced p*K*_w_ values observed in nanodroplet systems raise a broader question: To what extent are these changes driven by confinement itself?

**Fig. 4.**
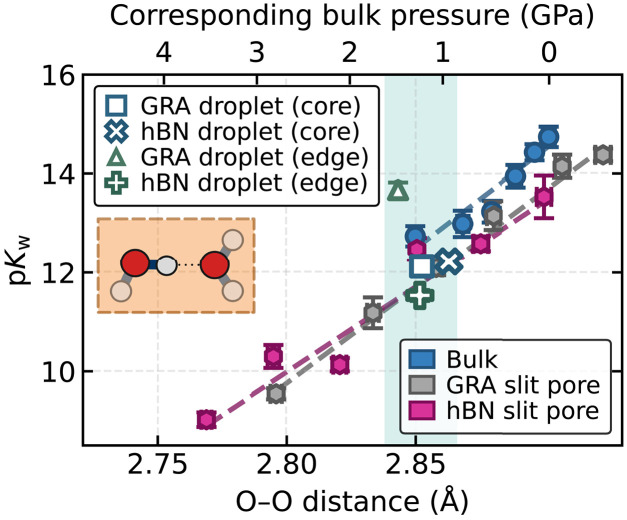
Unifying bulk and confined dissociation thermodynamics and outlining routes to modulation. Relationship between p*K*_w_ and the average O─O distances in O−H⋯O pairs, which promote water self-dissociation. The O─O distances are calculated either at the droplet core or near the edge, depending on where the dissociated species are located in each case (see section S7). The corresponding bulk pressure is indicated on the secondary (top) axis. The teal-shaded region highlights results for nanodroplets across different scenarios and indicates a corresponding bulk pressure of ∼1 GPa. The accompanying schematic illustrates a representative O−H⋯O pair.

To explore this, we turn to a microscopic structural descriptor that reflects a system’s ability to support proton transfer, a key step in self-dissociation. Specifically, we examine the average O─O distance in hydrogen-bonded O−H⋯O pairs, which couples strongly with the proton transfer barrier modulating how readily protons can be transferred. Figure 4 presents these distances across all systems studied and offers a unified view of how structural changes relate to dissociation behavior under both bulk and confined conditions.

As shown in Fig. 4, in most cases—whether in bulk water, slit pores, or nanodroplets—the variation in p*K*_w_ is well captured by changes in O−H⋯O distances. Although absolute O─O values vary slightly between systems, they exhibit parallel trends, indicating a common structural response to compression. This behavior mirrors the well-known effect of pressure in bulk water, where shorter O─O distances are associated with enhanced dissociation (*43*). Moreover, it aligns with the thermodynamic trends in Fig. 2B, where comparable shifts in chemical potential led to similar reductions in p*K*_w_ across environments. Notably, the degree of structural compression observed in nanodroplet cores corresponds to effective pressures approaching 1 GPa, consistent with earlier estimates (*10*, *55*), further reinforcing this pressure-like interpretation of confinement and directly accounting for the enhanced dissociation observed in these systems. Together, these observations suggest that confinement enhances dissociation through a shared mechanism: compression-induced shortening of hydrogen bonds that facilitates proton transfer. In addition to their mechanistic relevance, the O─O distances also serve as a practical descriptor for reactivity. Unlike full thermodynamic treatments such as the chemical potential–controlled comparisons presented earlier, O─O distances can be readily extracted from both simulations and experiments, offering a physically interpretable and broadly applicable proxy for dissociation behavior across environments. Crucially, however, the relationship between compression and dissociation is not universal. While shorter O─O distances generally correlate with increased proton transfer; this trend can be modulated by local interfacial properties, which alter the hydrogen-bonding environment and the stability of dissociated species. For example, at the edges of graphene and hBN nanodroplets, dissociation behavior may begin to diverge from bulk-like trends, influenced by localized interfacial effects such as strain or reactive surface sites that can stabilize dissociated species. In such cases, even subtle changes to the local environment can shift dissociation equilibria, highlighting how interfacial design can offer a route for tuning acid-base behavior at the nanoscale.

## DISCUSSION

Our results reframe how nanoscale aqueous reactivity should be interpreted. Rather than treating confinement as a standalone variable, we show that water reactivity depends critically on the thermodynamic state and interfacial context, a perspective enabled by adopting chemical potential as the central lens for comparison. This shift in framing not only reconciles previous contradictions but also lays the groundwork for more predictive control of confined water chemistry. These findings show that geometric confinement alone does not inherently alter water’s dissociation behavior. In particular, when the confined phase is in thermodynamic equilibrium with the bulk, the dissociation constant remains close to the bulk value, indicating that water self-dissociation is essentially insensitive to confinement, except in cases involving strong or specific interfacial interactions. In such cases, any apparent differences between confined and bulk systems reflect shifts in thermodynamic state or interfacial stabilization, rather than a change in the underlying dissociation mechanism. In practical terms, this implies that observed reactivity in experimental systems such as carbon membranes, nanocapillaries, or layered 2D materials may depend sensitively on local curvature, degree of hydration, or surface chemistry. Such sensitivity presents both a challenge in interpreting seemingly inconsistent measurements and an opportunity to deliberately tune reactivity through structural and environmental design.

This work addresses a long-standing ambiguity, where water self-dissociation under nanoconfinement has been reported as enhanced, suppressed, or unchanged relative to the bulk. Using machine-learned MD simulations trained at first-principles accuracy, we systematically explored dissociation equilibria across a diverse set of nanoconfined environments. By varying the chemical potential to enable thermodynamically consistent comparisons, we demonstrated that water confined within rigid graphene slit pores exhibits dissociation behavior similar to bulk water under equivalent conditions. This finding demystifies many prior discrepancies, which often stemmed from inconsistent reference states or uncontrolled differences in pressure and density. More generally, apparent contradictions across the literature can often be traced to differences in how the confined reference state is defined and equilibrated. In particular, variations in the underlying electronic-structure description and in the equilibration protocol used to set the confined water content can shift the effective thermodynamic state of the confined liquid. In addition, differences in what constitutes confinement itself can affect how results are interpreted (*56*, *57*), leading to differing conclusions about water self-dissociation under confinement. In this context, it is worth noting that alternative thermodynamic reference states for nanoconfined liquids have been proposed that avoid the use of an explicit reservoir, for example, by identifying confined states at which the pressure tensor becomes isotropic (*58*). Such an approach is particularly useful for multilayer confinement (3L and beyond), where the precise definition of the confined height has only a weak influence on the computed pressure and a meaningful scalar pressure can be robustly identified. In the present work, we adopt a chemical-potential framework, which provides a complementary route for defining thermodynamically consistent reference states and enables comparisons across confined systems without explicitly specifying a confined volume or pressure.

At the same time, our results reveal clear, physically grounded pathways through which confinement can modulate reactivity, especially when interfacial chemistry and structural heterogeneity are present. In encapsulated nanodroplets, an experimentally realizable form of confinement that introduces interfacial curvature and spatial heterogeneity, we identify a dissociative pathway at hBN interfaces in which hydroxide ions are stabilized via chemisorption at droplet edges. This mechanism lowers the free energy cost of dissociation, unlocking reactivity inaccessible in bulk water or chemically inert environments. More broadly, even chemically inert surfaces can substantially influence water behavior under confinement by reshaping the local solvation environment and hydrogen-bond network (*59*). This view is consistent with studies of nanoconfined water in materials such as mackinawite, which report pronounced confinement-induced changes in proton (*60*) and proton-hole (*61*) solvation and dynamics. These effects could be further enhanced by external electric fields, offering an additional design lever for tuning water dissociation at the nanoscale (*41*, *62*).

Rather than offering a single answer to how reactive water is at the nanoscale, we provide a unifying perspective that explains when and why dissociation is affected. These insights open possibilities for controlling aqueous reactivity in applications ranging from ion transport in energy storage to proton-mediated processes in catalysis and sensing. More broadly, understanding how reactive species can be selectively stabilized in confined environments lays the foundation for interface-design strategies that combine geometric control with chemical specificity to tune water chemistry at the nanoscale.

## MATERIALS AND METHODS

### Machine learning potentials

The MLPs used in this work were developed using the MACE architecture (*63*), using 128 invariant channels, two layers, and a cutoff distance of 6 Å. Each model captures semilocal interactions through an effective receptive field, which extends to 12 Å, corresponding to the product of the number of layers and the cutoff distance per layer. All models were trained to reproduce the revPBE-D3 reference potential energy surface with high fidelity (see section S2) (*41*). We developed two separate MLPs: one for graphene-water systems and one for hBN-water systems.

The graphene-water MLP is based on training data from (*64*), which includes water self-dissociated configurations across various density regimes within graphene confinement, including the ultraconfined limit. It also incorporates data from (*65*), covering interfaces from planar graphene to highly confined water in carbon nanotubes of varying radii. Together, these datasets allow the model to accurately capture nanoconfined water behavior in slit pores while also describing graphene’s bending rigidity, which is a key property for modeling nanodroplet formation (see section S2). To improve the description of graphene-graphene interactions, we included structures at equilibrium and slightly perturbed interlayer distances, for both AA and AB stacking. This ensures that the model properly describes the vdW-driven closure of the nanodroplet, which is governed by the attractive forces between the graphene sheets. Given the broad density range explored in this work, we incorporated representative high- and low-density configurations into the training set, including corresponding structures under rigid graphene confinement to ensure transferability across relevant thermodynamic conditions. We also varied the graphene layer separations to capture water behavior across distinct interlayer environments. Last, to reflect the large-scale systems considered here, we extended the training set with configurations from larger confined water systems. The final training set comprised 5845 structures, yielding root mean square errors of 0.9 meV per atom for energies and 26.3 meV/Å for forces.

We developed the hBN-water MLP using the same procedure, with additional training data from (*54*) to accurately describe OH^−^-hBN interactions. As with the graphene model, this MLP includes configurations from both slit pore and nanodroplet geometries, ensuring broad coverage of structural and thermodynamic conditions relevant to this study. The final training set comprised 5395 structures, yielding root mean square errors of 0.7 meV per atom for energies and 21.1 meV/Å for forces.

### Electronic structure

All electronic structure calculations to train the MLP were carried out using the CP2K/Quickstep code (*66*). The revPBE-D3 functional (*67*, *68*) was chosen for its reliable performance in capturing the structure and dynamics of liquid water (*69*–*71*) and its ionized products (*64*, *72*) as well as the interaction between water and graphene (*73*). Atomic cores were represented using dual-space Goedecker-Teter-Hutter pseudopotentials (*74*). In the Gaussian and plane waves method, the Kohn-Sham orbitals of oxygen and hydrogen atoms were represented using the TZV2P basis set, whereas carbon, nitrogen, and boron atoms were described using the DZVP basis set. The electron density was represented using an auxiliary plane-wave basis with a cutoff energy of 1050 Ry. See section S2 for further details.

### MD simulations

All simulations were performed using machine-learned potentials. Except for the large-scale simulations described at the end of this section, all simulations were carried out using the ASE software package (*75*), with enhanced sampling implemented via PLUMED (*76*). Dynamics were propagated at a temperature of 300 K in the NVT ensemble, with a 0.5-fs time step and a Langevin thermostat with a friction coefficient of 2.5 ps^−1^. All systems were modeled within orthorhombic simulation cells, applying periodic boundary conditions along all three spatial dimensions. All simulations used hydrogen atom masses. To avoid interactions between periodic images, a 15-Å vacuum, greater than the model’s receptive field, was introduced along the *z* axis.

Each of the p*K*_w_ values reported in this work was obtained from the free energy profiles for the water self-dissociation reaction via umbrella sampling (see section S3). In each umbrella window, the coordination number of a selected oxygen atom (O^*^) with all hydrogen atoms in the system was defined as

$$n_{H}=\sum_{i=1}^{N}\frac{1-{\left(r_{i}/R_{0}\right)}^{12}}{1-{\left(r_{i}/R_{0}\right)}^{24}}$$ (1)

where *i* iterates over all the hydrogens in the simulation box, *r_i_* is the distance between hydrogen *i* and O^*^, and *R*_0_ is a switching distance set to 1.38 Å from (*40*). To enforce sampling along the reaction coordinate, *n*_H_ was restrained around a target value $n_{H}^{\prime }$ using a harmonic potential with a force constant of 200 kcal/mol per coordination unit squared. A total of 31 windows with $n_{H}^{\prime }=1.00,1.04,\ldots ,2.20$ were considered for each energy profile. For each window, a simulation of 100 ps was performed. To reconstruct the free energy profile, umbrella integration (*77*) was used. In total, 27 p*K*_w_ estimates are reported in this work, each based on 3.1 ns of simulation time, amounting to over 80 ns of total simulation. This highlights the critical role of machine learning–based MD in making such extensive sampling computationally feasible. Because our goal is to understand how confinement modulates water dissociation, we emphasize relative differences in p*K*_w_ across systems rather than precise absolute values. This strategy ensures that our conclusions remain robust and transferable, as they are less sensitive to the choice of electronic structure method, neglect of nuclear quantum effects, or the specific sampling protocol.

To establish a chemical potential reference for nanoconfined water, allowing direct comparison with bulk water, we determined the equilibrium density of water confined within a rigid graphene slit pore immersed in an aqueous liquid reservoir. These simulations were performed in the NPT ensemble, maintaining a pressure of 1 bar. To preserve the structural rigidity of the graphene sheets, pressure control was applied anisotropically by allowing the simulation box to fluctuate only along the *x* direction. During volume changes, only the water molecules were rescaled, while the graphene sheets were kept fully frozen. No pressure coupling was applied along the *y* axis, which remained fully periodic. The system consisted of two parallel graphene layers of equal length (see Fig. 2A), and we computed the surface density within the central region of the slit pore to characterize the confined water. To obtain the equilibrium confined density, we systematically varied the size of the slit pores. For each pore size, the graphene sheets were immersed in a sufficiently large water reservoir, with the amount of surrounding water adjusted to ensure bulk-like behavior at the lateral edges of the graphene layers. We determined the equilibrium density in the confined system by extrapolating the densities obtained from these simulations (see section S5). To overcome the computational cost associated with these large-scale simulations, ranging from 15,000 to 30,000 atoms, we used the Symmetrix library (*78*, *79*), an optimized C++ and Kokkos implementation that accelerates machine-learned potentials for efficient large-scale inference. Symmetrix interfaces directly with LAMMPS (*80*), providing efficient large-scale inference.

## Supplementary Materials

### This PDF file includes:

Sections S1 to S8 Tables S1 to S11 Figs. S1 to S17 References
