# Supplementary material for: How reactive is water at the nanoscale and how to control it?
Source: Sci Adv. 2026 Jun 24;12(26):eaeb5772. doi: 10.1126/sciadv.aeb5772 (PMC13292949; doi:10.1126/sciadv.aeb5772)
Supplement: Supplementary file 1 — Sections S1 to S8 Tables S1 to S11 Figs. S1 to S17 References [file sciadv.aeb5772_sm.pdf]

Science Advances
AAAS

Supplementary Materials for

How reactive is water at the nanoscale and how to control it?

Xavier R. Advincula *et al.*

Corresponding author: Christoph Schran, cs2121@cam.ac.uk; Angelos Michaelides, am452@cam.ac.uk

Sci. Adv. **12**, eaeb5772 (2026)
DOI: 10.1126/sciadv.aeb5772

This PDF file includes:

Sections S1 to S8
Tables S1 to S11
Figs. S1 to S17
References

1 Molecular dynamics simulations

1.1 System setup

The systems investigated in this work span both bulk water and a range of nanoconfined environments. These include water confined between parallel rigid graphene (GRA) sheets, water confined between hexagonal boron nitride (hBN) sheets, and water encapsulated within graphene and hBN nanodroplets. In addition, we considered graphene slit pores immersed in an aqueous liquid reservoir, used to establish a chemical potential reference for confined water.

All simulations used orthorhombic cells with periodic boundary conditions in all three dimensions. In graphene-containing systems, the graphene sheets were constructed by repeating the unit cell dimensions $a = \sqrt{3}d_c$ and $b = 3d_c$ along the x and y directions, respectively, where $d_c = 1.42$ Å is the carbon–carbon bond length (*82*). For example, the graphene dimensions of $L_x = 44.460$ Å and $L_y = 47.058$ Å were obtained by repeating the unit cell 18 times along x and 11 times along y. This tiling of the unit cell ensures that the graphene sheet maintains its characteristic hexagonal lattice structure with a consistent carbon-carbon bond distance throughout the extended sheet. An analogous approach was applied to construct the hBN sheets.

A vacuum space of 15 Å was added in the z direction to prevent interactions between the periodic images in the confined systems, as this exceeds the model's receptive field. An overview of the systems investigated in this work is presented in Tables S1–S7, which contain all relevant information for these systems, as well as illustrative schematics.

**System**<br>**(dimensions)**	**Simulation details**	**Illustration**
Bulk water<br>(19.86 Å × 19.86 Å × 19.86 Å)	$N_{\text{atoms}} = 768$<br>$N_{\text{H}_2\text{O}} = 256$<br>$N_{\text{umbrellas}} = 31$<br>$t_{\text{eq}} = 50$ ps<br>$t_{\text{sim/umbrella}} = 100$ ps<br>$\rho_{\text{O}} = 0.9777$ g/cm^3<br>$p\text{K}_{\text{w}} = 14.72 \pm 0.21$<br>$\mu - \mu_0 = 0$ kJ/mol	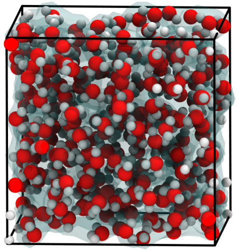
Bulk water<br>(19.60 Å × 19.60 Å × 19.60 Å)	$N_{\text{atoms}} = 768$<br>$N_{\text{H}_2\text{O}} = 256$<br>$N_{\text{umbrellas}} = 31$<br>$t_{\text{eq}} = 50$ ps<br>$t_{\text{sim/umbrella}} = 100$ ps<br>$\rho_{\text{O}} = 1.0171$ g/cm^3<br>$p\text{K}_{\text{w}} = 14.42 \pm 0.16$<br>$\mu - \mu_0 = 1.71$ kJ/mol	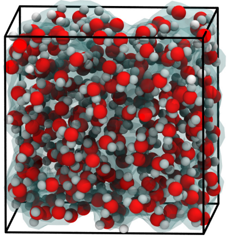
Bulk water<br>(19.24 Å × 19.24 Å × 19.24 Å)	$N_{\text{atoms}} = 768$<br>$N_{\text{H}_2\text{O}} = 256$<br>$N_{\text{umbrellas}} = 31$<br>$t_{\text{eq}} = 50$ ps<br>$t_{\text{sim/umbrella}} = 100$ ps<br>$\rho_{\text{O}} = 1.0753$ g/cm^3<br>$p\text{K}_{\text{w}} = 13.93 \pm 0.23$<br>$\mu - \mu_0 = 4.81$ kJ/mol	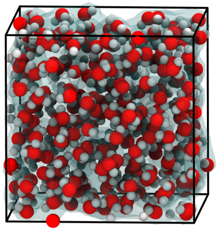

Bulk water<br>(18.93 Å × 18.93 Å × 18.93 Å)	$N_{\text{atoms}} = 768$<br>$N_{\text{H}_2\text{O}} = 256$<br>$N_{\text{umbrellas}} = 31$<br>$t_{\text{eq}} = 50$ ps<br>$t_{\text{sim/umbrella}} = 100$ ps<br>$\rho = 1.1290$ g/cm^3<br>$p\text{K}_\text{w} = 13.24 \pm 0.22$<br>$\mu - \mu_0 = 8.43$ kJ/mol	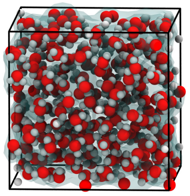
Bulk water<br>(18.61 Å × 18.61 Å × 18.61 Å)	$N_{\text{atoms}} = 768$<br>$N_{\text{H}_2\text{O}} = 256$<br>$N_{\text{umbrellas}} = 31$<br>$t_{\text{eq}} = 50$ ps<br>$t_{\text{sim/umbrella}} = 100$ ps<br>$\rho_\text{O} = 1.1882$ g/cm^3<br>$p\text{K}_\text{w} = 12.99 \pm 0.26$<br>$\mu - \mu_0 = 12.89$ kJ/mol	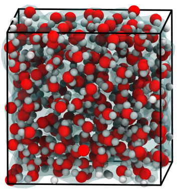
Bulk water<br>(18.20 Å × 18.20 Å × 18.20 Å)	$N_{\text{atoms}} = 768$<br>$N_{\text{H}_2\text{O}} = 256$<br>$N_{\text{umbrellas}} = 31$<br>$t_{\text{eq}} = 50$ ps<br>$t_{\text{sim/umbrella}} = 100$ ps<br>$\rho_\text{O} = 1.2704$ g/cm^3<br>$p\text{K}_\text{w} = 12.74 \pm 0.20$<br>$\mu - \mu_0 = 20.36$ kJ/mol	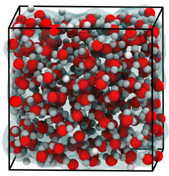

Table S1: **Detailed overview of the bulk systems considered in this work.** For each system, we report the total number of atoms, N_{atoms}; the corresponding number of water molecules, $N_{\text{H}_2\text{O}}$; the number of umbrellas sampled $N_{\text{umbrellas}}$; the equilibration time, t_{eq}; the simulation production time per umbrella, $t_{\text{sim/umbrella}}$; its density, ρ_O; its $p\text{K}_\text{w}$ value; and the chemical potential difference relative to the reference, $\mu - \mu_0$.

**System (dimensions)**	**Simulation details**	**Illustration**
Monolayer water confined between parallel rigid GRA sheets (44.460 Å × 47.058 Å × 21.700 Å)	$N_{\text{atoms}} = 2214$<br>$N_{\text{C}} = 1584$<br>$N_{\text{H}_2\text{O}} = 210$<br>$N_{\text{umbrellas}} = 31$<br>$t_{\text{eq}} = 50$ ps<br>$t_{\text{sim/umbrella}} = 100$ ps<br>$\rho_{\text{O}}^{\text{2D}} = 0.1003$ #O/Å^2<br>$p\text{K}_{\text{w}} = 14.36 \pm 0.13$<br>$\mu - \mu_0 = 0.00$ kJ/mol	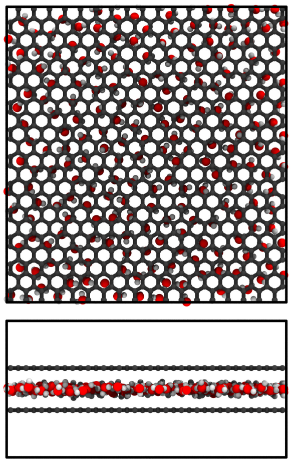
Monolayer water confined between parallel rigid GRA sheets (44.460 Å × 47.058 Å × 21.700 Å)	$N_{\text{atoms}} = 2250$<br>$N_{\text{C}} = 1584$<br>$N_{\text{H}_2\text{O}} = 222$<br>$N_{\text{umbrellas}} = 31$<br>$t_{\text{eq}} = 50$ ps<br>$t_{\text{sim/umbrella}} = 100$ ps<br>$\rho_{\text{O}}^{\text{2D}} = 0.1062$ #O/Å^2<br>$p\text{K}_{\text{w}} = 14.13 \pm 0.23$<br>$\mu - \mu_0 = 1.55$ kJ/mol	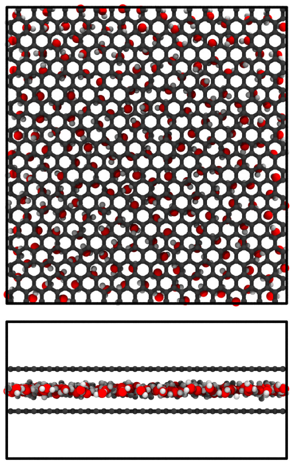
Monolayer water confined between parallel rigid GRA sheets (44.460 Å × 47.058 Å × 21.700 Å)	$N_{\text{atoms}} = 2289$<br>$N_{\text{C}} = 1584$<br>$N_{\text{H}_2\text{O}} = 235$<br>$N_{\text{umbrellas}} = 31$<br>$t_{\text{eq}} = 50$ ps<br>$t_{\text{sim/umbrella}} = 100$ ps<br>$\rho_{\text{O}}^{\text{2D}} = 0.1121$ #O/Å^2<br>$p\text{K}_{\text{w}} = 13.15 \pm 0.28$<br>$\mu - \mu_0 = 8.83$ kJ/mol	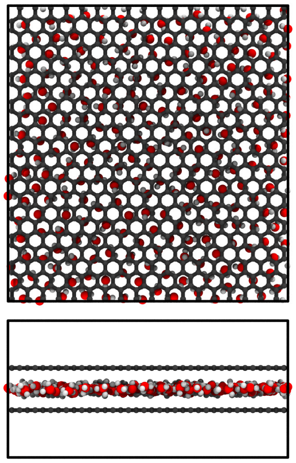

Monolayer water confined between parallel rigid GRA sheets (44.460 Å × 47.058 Å × 21.700 Å)	$N_{\mathrm{atoms}} = 2325$<br>$N_{\mathrm{C}} = 1584$<br>$N_{\mathrm{H_2O}} = 247$<br>$N_{\mathrm{umbrellas}} = 31$<br>$t_{\mathrm{eq}} = 50$ ps<br>$t_{\mathrm{sim/umbrella}} = 100$ ps<br>$\rho_{\mathrm{O}}^{\mathrm{2D}} = 0.1180$ #O/Å^2<br>$p\mathrm{K_w} = 12.08 \pm 0.16$<br>$\mu - \mu_0 = 17.91$ kJ/mol	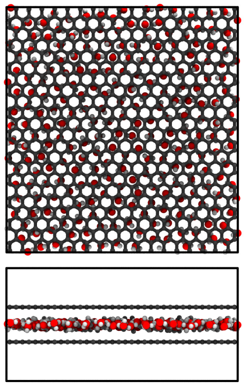
Monolayer water confined between parallel rigid GRA sheets (44.460 Å × 47.058 Å × 21.700 Å)	$N_{\mathrm{atoms}} = 2361$<br>$N_{\mathrm{C}} = 1584$<br>$N_{\mathrm{H_2O}} = 259$<br>$N_{\mathrm{umbrellas}} = 31$<br>$t_{\mathrm{eq}} = 50$ ps<br>$t_{\mathrm{sim/umbrella}} = 100$ ps<br>$\rho_{\mathrm{O}}^{\mathrm{2D}} = 0.1239$ #O/Å^2<br>$p\mathrm{K_w} = 11.17 \pm 0.31$<br>$\mu - \mu_0 = 28.61$ kJ/mol	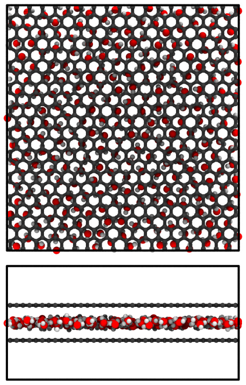
Monolayer water confined between parallel rigid GRA sheets (44.460 Å × 47.058 Å × 21.700 Å)	$N_{\mathrm{atoms}} = 2400$<br>$N_{\mathrm{C}} = 1584$<br>$N_{\mathrm{H_2O}} = 272$<br>$N_{\mathrm{umbrellas}} = 31$<br>$t_{\mathrm{eq}} = 50$ ps<br>$t_{\mathrm{sim/umbrella}} = 100$ ps<br>$\rho_{\mathrm{O}}^{\mathrm{2D}} = 0.1298$ #O/Å^2<br>$p\mathrm{K_w} = 9.54 \pm 0.11$<br>$\mu - \mu_0 = 42.03$ kJ/mol	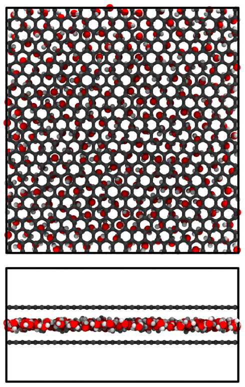

Table S2: **Detailed overview of the monolayer confined water systems between parallel graphene layers considered in this work.** For each system, we report the total number of atoms, N_{atoms}; the number of carbon atoms, N_{C}; the corresponding number of water molecules, $N_{\mathrm{H_2O}}$; the number of umbrellas sampled $N_{\mathrm{umbrellas}}$; the equilibration time, t_{eq}; the simulation production time per umbrella, $t_{\mathrm{sim/umbrella}}$; its surface density, $\rho_{\mathrm{O}}^{\mathrm{2D}}$ (total number of oxygen atoms per lateral area of the confining surfaces); its $p\mathrm{K_w}$ value; and the chemical potential difference relative to the reference, $\mu - \mu_0$.

**System (dimensions)**	**Simulation details**	**Illustration**
Monolayer water confined between parallel rigid GRA sheets (44.460 Å × 47.058 Å × 21.700 Å)	$N_{\text{atoms}} = 2214$<br>$N_{\text{C}} = 1584$<br>$N_{\text{H}_2\text{O}} = 210$<br>$N_{\text{umbrellas}} = 31$<br>$t_{\text{eq}} = 50$ ps<br>$t_{\text{sim/umbrella}} = 100$ ps<br>$\rho_{\text{O}}^{\text{2D}} = 0.1003$ #O/Å^2<br>$p\text{K}_{\text{w}} = 14.36 \pm 0.13$	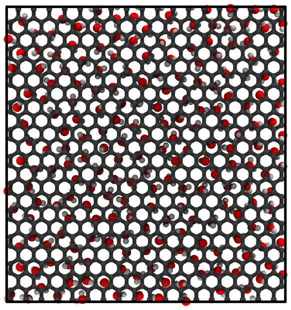<br>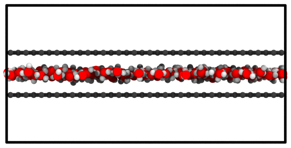
Bilayer water confined between parallel rigid GRA sheets (44.460 Å × 47.058 Å × 25.050 Å)	$N_{\text{atoms}} = 2844$<br>$N_{\text{C}} = 1584$<br>$N_{\text{H}_2\text{O}} = 420$<br>$N_{\text{umbrellas}} = 31$<br>$t_{\text{eq}} = 50$ ps<br>$t_{\text{sim/umbrella}} = 100$ ps<br>$\rho_{\text{O}}^{\text{2D}} = 0.2006$ #O/Å^2<br>$p\text{K}_{\text{w}} = 14.18 \pm 0.33$	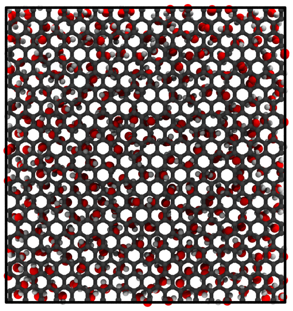<br>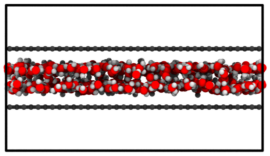
Trilayer water confined between parallel rigid GRA sheets (44.460 Å × 47.058 Å × 28.400 Å)	$N_{\text{atoms}} = 3474$<br>$N_{\text{C}} = 1584$<br>$N_{\text{H}_2\text{O}} = 630$<br>$N_{\text{umbrellas}} = 31$<br>$t_{\text{eq}} = 50$ ps<br>$t_{\text{sim/umbrella}} = 100$ ps<br>$\rho_{\text{O}}^{\text{2D}} = 0.3009$ #O/Å^2<br>$p\text{K}_{\text{w}} = 14.48 \pm 0.22$	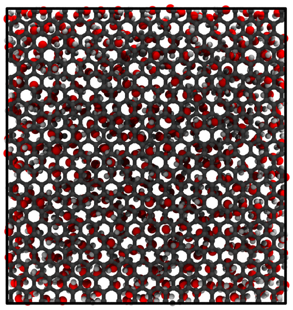<br>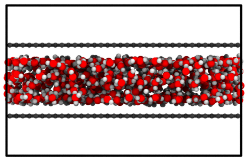

Monolayer water confined between parallel flexible GRA sheets (44.460 Å × 47.058 Å × 21.700 Å)	$N_{\text{atoms}} = 2214$<br>$N_{\text{C}} = 1584$<br>$N_{\text{H}_2\text{O}} = 210$<br>$N_{\text{umbrellas}} = 31$<br>$t_{\text{eq}} = 50$ ps<br>$t_{\text{sim/umbrella}} = 100$ ps<br>$\rho_{\text{O}}^{\text{2D}} = 0.1003\ \#\text{O}/\text{Å}^2$<br>$p\text{K}_{\text{w}} = 15.13 \pm 0.13$	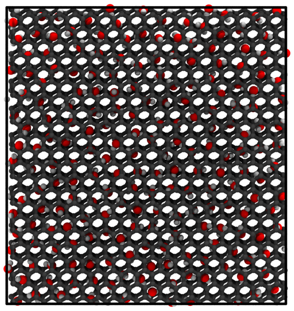<br>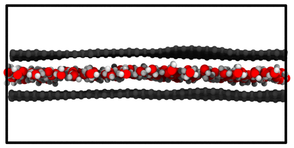
Bilayer water confined between parallel flexible GRA sheets (44.460 Å × 47.058 Å × 25.050 Å)	$N_{\text{atoms}} = 2844$<br>$N_{\text{C}} = 1584$<br>$N_{\text{H}_2\text{O}} = 420$<br>$N_{\text{umbrellas}} = 31$<br>$t_{\text{eq}} = 50$ ps<br>$t_{\text{sim/umbrella}} = 100$ ps<br>$\rho_{\text{O}}^{\text{2D}} = 0.2006\ \#\text{O}/\text{Å}^2$<br>$p\text{K}_{\text{w}} = 15.27 \pm 0.13$	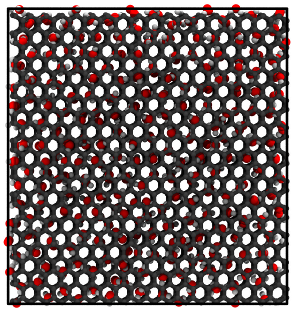<br>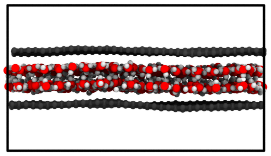
Trilayer water confined between parallel flexible GRA sheets (44.460 Å × 47.058 Å × 28.400 Å)	$N_{\text{atoms}} = 3474$<br>$N_{\text{C}} = 1584$<br>$N_{\text{H}_2\text{O}} = 630$<br>$N_{\text{umbrellas}} = 31$<br>$t_{\text{eq}} = 50$ ps<br>$t_{\text{sim/umbrella}} = 100$ ps<br>$\rho_{\text{O}}^{\text{2D}} = 0.3009\ \#\text{O}/\text{Å}^2$<br>$p\text{K}_{\text{w}} = 14.03 \pm 0.23$	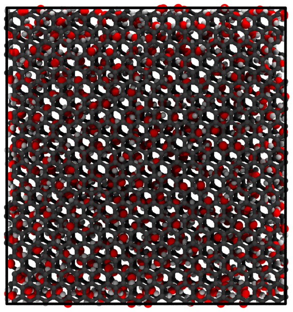<br>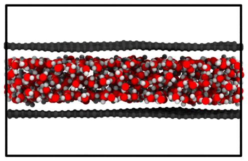

Table S3: **Detailed overview of the systems reported in Figure S4.** For each system, we report the total number of atoms, N_{atoms}; the number of carbon atoms, N_{C}; the corresponding number of water molecules, $N_{\mathrm{H_2O}}$; the number of umbrellas sampled $N_{\mathrm{umbrellas}}$; the equilibration time, t_{eq}; the simulation production time per umbrella, $t_{\mathrm{sim/umbrella}}$; its surface density, $\rho_{\mathrm{O}}^{\mathrm{2D}}$ (total number of oxygen atoms per lateral area of the confining surfaces); and its $p\mathrm{K_w}$ value. As specified in the main text, the rigid 1L, 2L, and 3L setups correspond to slit widths of 6.70, 10.05, and 13.40 Å, respectively. These values are commensurate with pore sizes that can be experimentally realized using vdW assembly (*13, 83*). In the bilayer setup, the number of water molecules was set to twice that of the monolayer case, based on the observation that two well-defined layers of water form at this width. A similar assumption was applied to the trilayer setup. It is important to note, however, that these configurations are used for illustrative purposes. As emphasized throughout the manuscript, rigorous comparisons require thermodynamic consistency across these systems. For example, direct comparisons between rigid and flexible pores are complicated by the fact that flexible systems, even when exhibiting a similar average interlayer spacing, can accommodate a wider range of densities and layering motifs (*42*).

System<br>(dimensions)	Simulation details	Illustration
Monolayer water confined between parallel rigid hBN sheets<br>(43.490 Å × 45.198 Å × 21.700 Å)	$N_{\mathrm{atoms}} = 2070$<br>$N_{\mathrm{B/N}} = 1440$<br>$N_{\mathrm{H_2O}} = 210$<br>$N_{\mathrm{umbrellas}} = 31$<br>$t_{\mathrm{eq}} = 50$ ps<br>$t_{\mathrm{sim/umbrella}} = 100$ ps<br>$\rho_{\mathrm{O}}^{\mathrm{2D}} = 0.1003\ \#\mathrm{O}/\mathrm{Å}^2$<br>$p\mathrm{K_w} = 13.52 \pm 0.43$	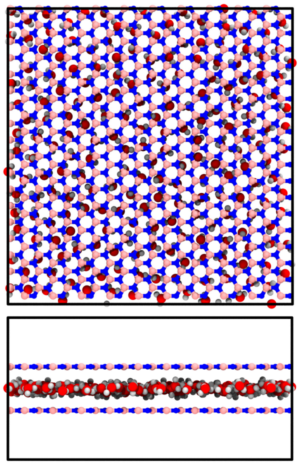
Monolayer water confined between parallel rigid hBN sheets<br>(43.490 Å × 45.198 Å × 21.700 Å)	$N_{\mathrm{atoms}} = 2106$<br>$N_{\mathrm{B/N}} = 1440$<br>$N_{\mathrm{H_2O}} = 222$<br>$N_{\mathrm{umbrellas}} = 31$<br>$t_{\mathrm{eq}} = 50$ ps<br>$t_{\mathrm{sim/umbrella}} = 100$ ps<br>$\rho_{\mathrm{O}}^{\mathrm{2D}} = 0.1062\ \#\mathrm{O}/\mathrm{Å}^2$<br>$p\mathrm{K_w} = 12.59 \pm 0.15$	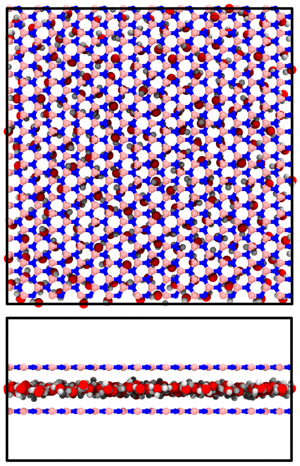
Monolayer water confined between parallel rigid hBN sheets<br>(43.490 Å × 45.198 Å × 21.700 Å)	$N_{\mathrm{atoms}} = 2145$<br>$N_{\mathrm{B/N}} = 1440$<br>$N_{\mathrm{H_2O}} = 235$<br>$N_{\mathrm{umbrellas}} = 31$<br>$t_{\mathrm{eq}} = 50$ ps<br>$t_{\mathrm{sim/umbrella}} = 100$ ps<br>$\rho_{\mathrm{O}}^{\mathrm{2D}} = 0.1121\ \#\mathrm{O}/\mathrm{Å}^2$<br>$p\mathrm{K_w} = 12.48 \pm 0.23$	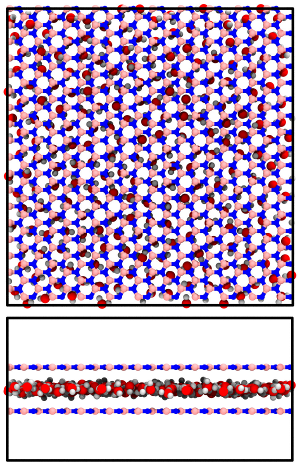

Monolayer water confined between parallel rigid hBN sheets (43.490 Å × 45.198 Å × 21.700 Å)	$N_{\mathrm{atoms}} = 2181$<br>$N_{\mathrm{B/N}} = 1440$<br>$N_{\mathrm{H_2O}} = 247$<br>$N_{\mathrm{umbrellas}} = 31$<br>$t_{\mathrm{eq}} = 50$ ps<br>$t_{\mathrm{sim/umbrella}} = 100$ ps<br>$\rho_{\mathrm{O}}^{\mathrm{2D}} = 0.1180$ #O/Å^2<br>$p\mathrm{K_w} = 10.13 \pm 0.13$	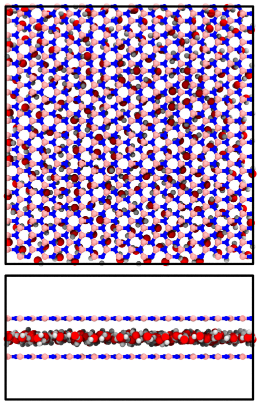
Monolayer water confined between parallel rigid hBN sheets (43.490 Å × 45.198 Å × 21.700 Å)	$N_{\mathrm{atoms}} = 2217$<br>$N_{\mathrm{B/N}} = 1440$<br>$N_{\mathrm{H_2O}} = 259$<br>$N_{\mathrm{umbrellas}} = 31$<br>$t_{\mathrm{eq}} = 50$ ps<br>$t_{\mathrm{sim/umbrella}} = 100$ ps<br>$\rho_{\mathrm{O}}^{\mathrm{2D}} = 0.1239$ #O/Å^2<br>$p\mathrm{K_w} = 10.29 \pm 0.09$	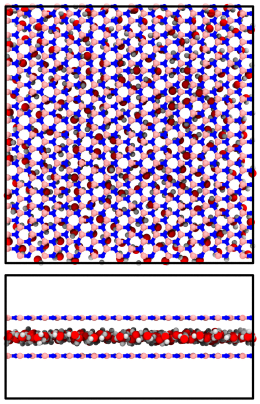
Monolayer water confined between parallel rigid hBN sheets (43.490 Å × 45.198 Å × 21.700 Å)	$N_{\mathrm{atoms}} = 2256$<br>$N_{\mathrm{B/N}} = 1440$<br>$N_{\mathrm{H_2O}} = 272$<br>$N_{\mathrm{umbrellas}} = 31$<br>$t_{\mathrm{eq}} = 50$ ps<br>$t_{\mathrm{sim/umbrella}} = 100$ ps<br>$\rho_{\mathrm{O}}^{\mathrm{2D}} = 0.1298$ #O/Å^2<br>$p\mathrm{K_w} = 9.02 \pm 0.15$	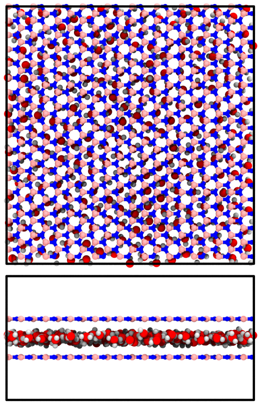

Table S4: **Detailed overview of the monolayer confined water systems between parallel hexagonal boron nitride layers considered in this work.** For each system, we report the total number of atoms, N_{atoms}; the number of boron and nitrogen atoms, $N_{\mathrm{B/N}}$; the corresponding number of water molecules, $N_{\mathrm{H_2O}}$; the number of umbrellas sampled $N_{\mathrm{umbrellas}}$; the equilibration time, t_{eq}; the simulation production time per umbrella, $t_{\mathrm{sim/umbrella}}$; its surface density, $\rho_{\mathrm{O}}^{\mathrm{2D}}$ (total number of oxygen atoms per lateral area of the confining surfaces); and its $p\mathrm{K_w}$ value.

**System (dimensions)**	**Simulation details**	**Illustration**
GRA nanodroplet confined water (79.040 Å × 81.282 Å × 40.000 Å)	$N_{\mathrm{atoms}} = 5260$<br>$N_{\mathrm{C}} = 4864$<br>$N_{\mathrm{H_2O}} = 132$<br>$N_{\mathrm{umbrellas}} = 31$<br>$t_{\mathrm{eq}} = 50$ ps<br>$t_{\mathrm{sim/umbrella}} = 100$ ps<br>$\rho_{\mathrm{O}}^{\mathrm{2D}} = 0.1180 \pm 0.0010$ #O/Å^2	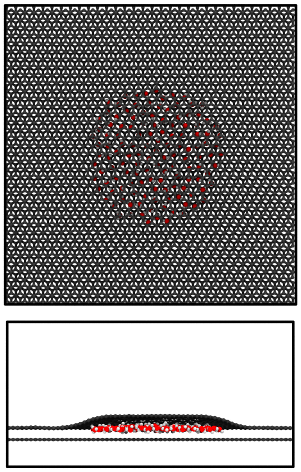

Table S5: **Detailed overview of the GRA nanodroplet confined water system considered in this work.** For the system, we report the total number of atoms, N_{atoms}; the number of carbon atoms, N_{C}; the corresponding number of water molecules, $N_{\mathrm{H_2O}}$; the number of umbrellas sampled $N_{\mathrm{umbrellas}}$; the equilibration time, t_{eq}; the simulation production time per umbrella, $t_{\mathrm{sim/umbrella}}$; and its radial density (see Section 6), $\rho_{\mathrm{O}}^{\mathrm{2D}}$.

System (dimensions)	Simulation details	Illustration
hBN nanodroplet confined water<br>(78.282 Å × 80.352 Å × 40.000 Å)	$N_{\text{atoms}} = 5004$<br>$N_{\text{B/N}} = 4608$<br>$N_{\text{H}_2\text{O}} = 132$<br>$N_{\text{umbrellas}} = 31$<br>$t_{\text{eq}} = 50$ ps<br>$t_{\text{sim/umbrella}} = 100$ ps<br>$\rho_{\text{O}}^{\text{2D}} = 0.1170 \pm 0.0010$ #O/Å^2	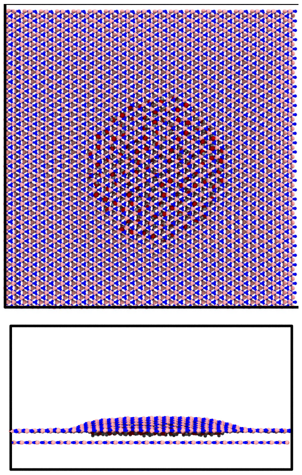

Table S6: **Detailed overview of the hBN nanodroplet confined water system considered in this work.** For the system, we report the total number of atoms, N_{atoms}; the number of boron and nitrogen atoms, $N_{\text{B/N}}$; the corresponding number of water molecules, $N_{\text{H}_2\text{O}}$; the number of umbrellas sampled $N_{\text{umbrellas}}$; the equilibration time, t_{eq}; the simulation production time per umbrella, $t_{\text{sim/umbrella}}$; and its radial density (see Section 6), $\rho_{\text{O}}^{\text{2D}}$.

**System**<br>**(avg. dimensions, since NPT)**	**Simulation details**	**Illustration**
Parallel rigid GRA layers<br>immersed in liquid water<br>(76.771 Å × 47.058 Å × 36.000 Å)	$N_{\mathrm{atoms}} = 13827$<br>$N_{\mathrm{C}} = 1584$<br>$a = 44.460$ Å<br>$b = 47.058$ Å<br>$N_{\mathrm{H_2O}} = 4081$<br>$W = 6.7$ Å<br>$t_{\mathrm{prod}} = 300$ ps<br>$t_{\mathrm{eq}} = 150$ ps<br>$\rho_{\mathrm{O}}^{\mathrm{2D}} = 0.1097$ #O/Å^2	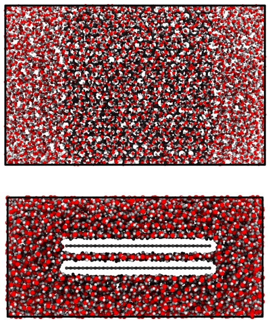
Parallel rigid GRA layers<br>immersed in liquid water<br>(86.762 Å × 55.614 Å × 36.000 Å)	$N_{\mathrm{atoms}} = 18208$<br>$N_{\mathrm{C}} = 2288$<br>$a = 54.340$ Å<br>$b = 55.614$ Å<br>$N_{\mathrm{H_2O}} = 5308$<br>$W = 6.7$ Å<br>$t_{\mathrm{prod}} = 300$ ps<br>$t_{\mathrm{eq}} = 150$ ps<br>$\rho_{\mathrm{O}}^{\mathrm{2D}} = 0.1083$ #O/Å^2	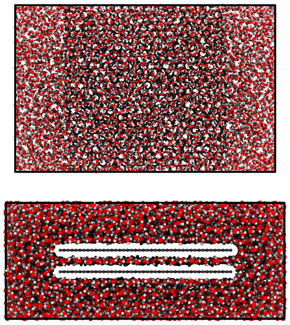
Parallel rigid GRA layers<br>immersed in liquid water<br>(96.694 Å × 64.170 Å × 36.000 Å)	$N_{\mathrm{atoms}} = 23051$<br>$N_{\mathrm{C}} = 3120$<br>$a = 64.220$ Å<br>$b = 64.170$ Å<br>$N_{\mathrm{H_2O}} = 6645$<br>$W = 6.7$ Å<br>$t_{\mathrm{prod}} = 300$ ps<br>$t_{\mathrm{eq}} = 150$ ps<br>$\rho_{\mathrm{O}}^{\mathrm{2D}} = 0.1066$ #O/Å^2	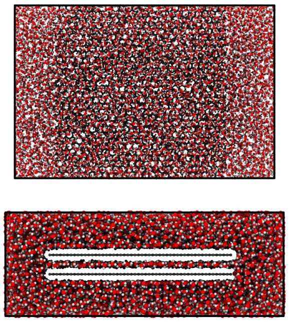

Parallel rigid GRA layers immersed in liquid water (105.742 Å × 77.004 Å × 36.000 Å)	$N_{\mathrm{atoms}} = 29971$<br>$N_{\mathrm{C}} = 4320$<br>$a = 74.100$ Å<br>$b = 77.004$ Å<br>$N_{\mathrm{H_2O}} = 8553$<br>$W = 6.7$ Å<br>$t_{\mathrm{prod}} = 300$ ps<br>$t_{\mathrm{eq}} = 150$ ps<br>$\rho_{\mathrm{O}}^{\mathrm{2D}} = 0.1061$ #O/Å^2	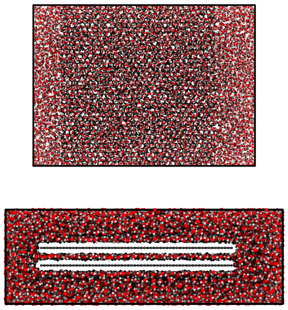

Table S7: **Detailed overview of the parallel rigid GRA layer systems immersed in liquid water, used in this work to establish a chemical potential reference.** For each system, we report the total number of atoms, N_{atoms}; the number of carbon atoms, N_{C}; the graphene in-plane lattice constant along the x-direction, a; the graphene in-plane lattice constant along the y-direction, b; the corresponding number of water molecules, $N_{\mathrm{H_2O}}$; the interlayer distance between the graphene sheets, W; the equilibration time, t_{eq}; the simulation production time per umbrella, $t_{\mathrm{sim/umbrella}}$; and the surface density within the central region of the slit, $\rho_{\mathrm{O}}^{\mathrm{2D}}$.

1.2 Simulation setup

In this work, we conducted five different types of molecular dynamics (MD) simulations: (i) short MD simulations using the machine-learned potentials (MLPs) to generate additional training data for its development; (ii) *ab initio* MD (AIMD) simulations to validate the MLP; (iii) constrained MD simulations using the developed MLP, which form the core of the results presented in this work; (iv) extended MD simulations using the developed MLP to obtain more accurate estimates of key observables; and (v) large-scale simulations using the MLP, where rigid slit pores are fully immersed in bulk liquid water, to determine the equilibrium surface density inside the central region of the pores. All simulations used hydrogen atom masses.

Short MD simulations

To ensure that the MLP was trained on the range of configurations sampled during the production runs, we expanded the training dataset beyond our base data (from Refs. 64 and 65). Specifically, we included configurations that accurately describe graphene–graphene interactions in both AA and AB stackings, as well as structures spanning a range of water densities, from high to low. For this purpose, we used the MLP to propagate dynamics via Langevin MD simulations in the NVT ensemble at 300 K, using a time step of 0.5 fs and a friction coefficient of 2.5 ps^{-1}. These simulations were carried out using the ASE software (*75*). A similar data generation and training procedure was used to develop a separate MLP tailored to water interacting with hBN in both slit pore and nanodroplet environments.

AIMD simulations

The AIMD simulations used to validate the MLP (see Section 2) were performed in the NVT ensemble using the CP2K/Quickstep code (*66*), interfaced with the i-PI program (*84*), with a time step of 1.0 fs. The temperature was set to 300 K, 330 K, 360 K, and 400 K, maintained using a CSVR thermostat (*85*) with a 100 fs coupling constant. We employed the revPBE generalized gradient approximation exchange-correlation functional (*67*), combined with the zero-damping version of Grimme's D3 dispersion correction (*68*). Atomic cores were represented using dual-space Goedecker–Teter–Hutter pseudopotentials (*74*), and a plane-wave cutoff of 400 Ry was applied. The Kohn–Sham orbitals of oxygen and hydrogen atoms were expanded using the TZV2P basis set (*86*).

Constrained MD simulations

The constrained MD simulations were performed using the ASE software (*75*) with the PLUMED plugin (*76*). Each umbrella window was propagated via Langevin MD simulations in the NVT ensemble at 300 K, using a time step of 0.5 fs and a friction coefficient of 2.5 ps^{-1}. For each window, a 50 ps equilibration period was followed by a 100 ps production period, from which statistics of the umbrellas were obtained.

Extended MD simulations

To obtain further microscopic insights into the systems analysed, extended (unconstrained) MD simulations were performed using the ASE software (*75*). For this, a 50 ps equilibration period was followed by a 1 ns production period, from which statistics of the properties of interest were obtained (mainly pressure, distance, and hydrogen bonding statistics). The dynamics were propagated via

Langevin MD simulations in the NVT ensemble at 300 K, using a time step of 0.5 fs and a friction coefficient of 2.5 ps^{-1}

Large-scale MD simulations

To determine the equilibrium surface density within the central region of slit pores –as required for setting a reference chemical potential (see Section 5)– we performed large-scale molecular dynamics simulations using the Symmetrix library (*78, 79*), which interfaces directly with LAMMPS (*80*). The systems consisted of rigid slit pores fully immersed in bulk liquid water. Simulations were conducted in the NPT ensemble at 300 K with a 1 fs time step. Each system was equilibrated for 150 ps, followed by a 300 ps production run, during which the surface density in the central pore region was measured. Pressure control was applied anisotropically by allowing the simulation box to fluctuate only along the x-direction, with the remaining dimensions held fixed to reflect confinement. Only the water molecules were rescaled during volume changes, while the graphene sheets were kept fully frozen.

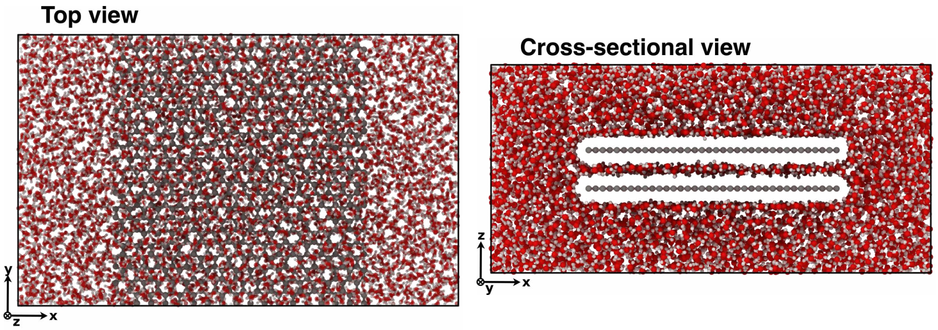

Figure S1: **Top and cross-sectional views of the simulation setup used to equilibrate confined water, showing the confining sheets, surrounding water, and the corresponding reference axes.**

2 Machine learning potential

2.1 Model development

The MLP was developed iteratively over several generations, beginning with training data from Refs. 64 and 65, selected to provide broad coverage of the relevant physical regimes. These datasets provide (i) self-dissociated water configurations across a wide range of densities under graphene confinement, including the ultra-confined limit, and (ii) water–graphene interfaces spanning from flat sheets to highly curved environments within carbon nanotubes of varying radii. Together, they ensure that the model captures both the physics of water dissociation and the bending rigidity of graphene—an essential feature for accurately modeling nanodroplet formation.

To refine the description of graphene–graphene interactions, we introduced structures corresponding to the equilibrium interlayer distance and included variations across both AA and AB stacking configurations. This was critical for capturing the van der Waals interactions that drive the graphene encapsulation of water. Given the range of densities (and, effectively, pressures) sampled in this work, we enriched the dataset with both high- and low-density configurations. We also incorporated water confined between AB-stacked graphene layers, as this stacking is key to the geometry of the encapsulated nanodroplet system shown in Figure 1A. Finally, we extended the training data to include configurations with expanded graphene sheet dimensions to ensure the model's applicability to the large-scale systems considered here. This comprehensive dataset allows the MLP to robustly describe the full range of thermodynamic and structural conditions explored in this work.

The MLP for hBN nanodroplets was developed following the same iterative strategy, with the addition of configurations from Ref. 54 to accurately capture water-hBN interactions specific to those environments.

2.2 Model validation

To evaluate the validity of the MLP for the systems studied in this work, we quantified the root-mean-square errors (RMSEs) in energies and forces predicted by the model. Specifically, we randomly selected 300 snapshots from 100 ps MLP-based MD simulations that sample the water

self-dissociation reaction pathway in either bulk or confined conditions, using values of n_{H} = $1.00, 1.20, \ldots, 2.00$. For each configuration, we performed single-point DFT calculations at the same level of theory used to train the MLP (i.e., revPBE-D3). This provides a direct and robust measure of the MLP's accuracy, as it compares predictions for structures sampled from its potential energy surface against the reference *ab initio* values.

To reduce the computational cost of these electronic structure calculations, we used smaller simulation cells. For graphene, the sheet dimensions were set to $L_x = 12.350$ Å and $L_y = 12.834$ Å. For hBN, we similarly employed reduced sheet dimensions of $L_x = 13.047$ Å and $L_y = 12.555$ Å. As shown in Figs. S2–S4, the MLP closely reproduces both the energies and forces obtained from the underlying DFT method across all systems and reaction coordinate values. This confirms the model's reliability in capturing the key physicochemical features relevant to water self-dissociation in the environments considered.

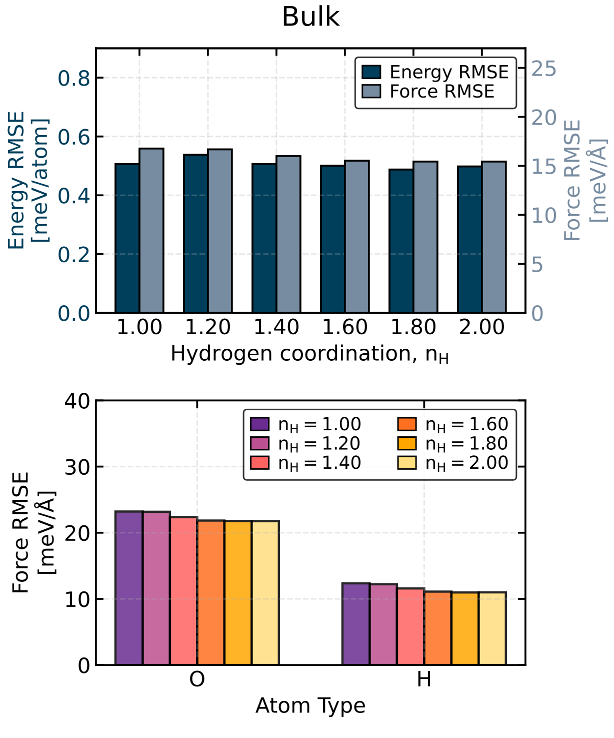

Figure S2: **RMSE in energies and forces along the water self-dissociation reaction pathway in the bulk setup, comparing the MLP predictions to the underlying DFT reference.** The force RMSE decomposed by atom type (O and H) along the reaction coordinate is also shown, highlighting the model's accuracy across all atomic species throughout the dissociation process.

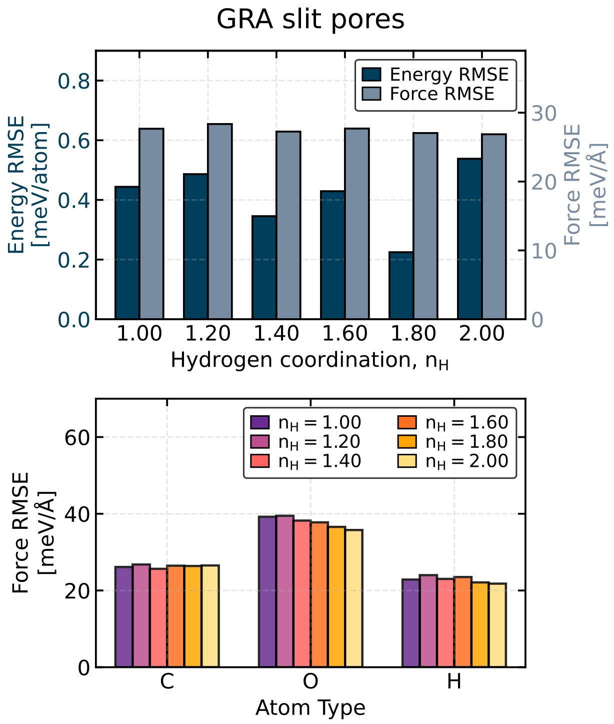

Figure S3: **RMSE in energies and forces along the water self-dissociation reaction pathway in the GRA slit pores, comparing the MLP predictions to the underlying DFT reference.** The force RMSE decomposed by atom type (C, O, and H) along the reaction coordinate is also shown, highlighting the model's accuracy across all atomic species throughout the dissociation process.

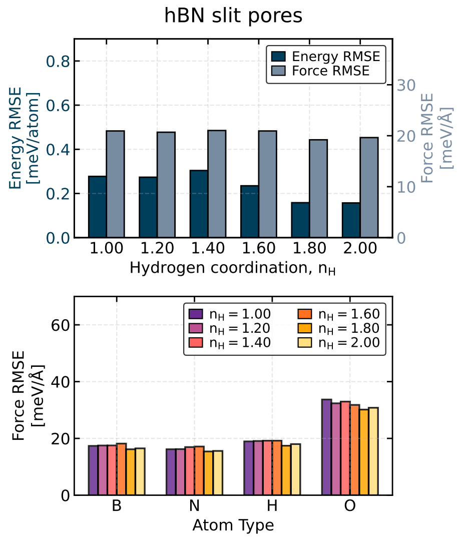

Figure S4: **RMSE in energies and forces along the water self-dissociation reaction pathway in the hBN slit pores, comparing the MLP predictions to the underlying DFT reference.** The force RMSE decomposed by atom type (B, N, O, and H) along the reaction coordinate is also shown, highlighting the model's accuracy across all atomic species throughout the dissociation process.

To further validate the robustness of the MLP developed in this work, we assessed its ability to reproduce the temperature dependence of the water self-dissociation reaction. While a focus of this study is on how water self-dissociation varies with density, accurately reproducing the thermodynamic response over a broader temperature range is a strong indicator of model transferability and physical fidelity. As shown in Figure S5, the free energy barriers computed using MACE closely match those obtained from AIMD across temperatures (*41*), with excellent agreement in the extracted enthalpic and entropic contributions. This consistency demonstrates that the MLP not only reproduces accurate energetics but also captures the underlying thermodynamic landscape of the dissociation process.

Finally, because a central focus of this work is the self-dissociation of water in material-encapsulated nanodroplets, it is essential that the model accurately captures the bending rigidity of the confining material, which determines how the material deforms to accommodate the droplet shape. For graphene, the bending rigidity, B_M, can be obtained by fitting the energy per atom in

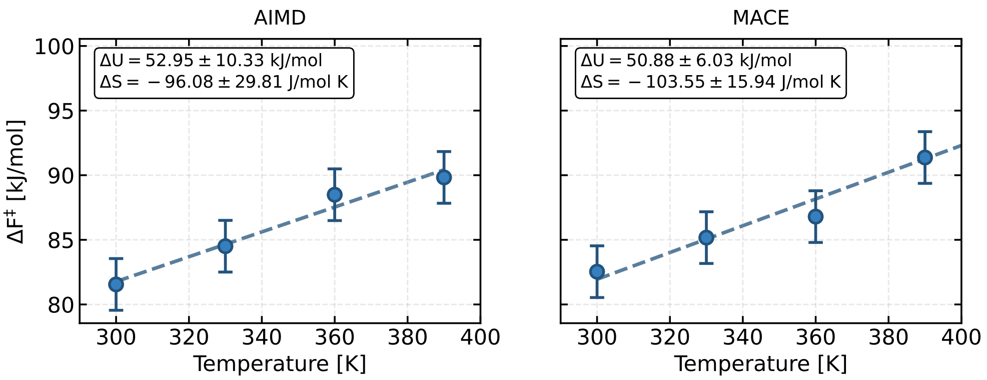

Figure S5: **Comparison of temperature-dependent free energy barriers for water self-dissociation computed using AIMD (*41*) and MACE MLP simulations.** The free energy barrier, $\Delta F^{\ddagger}$, is shown as a function of temperature T for AIMD (left) and MACE (right), and fitted using the relation $\Delta F = \Delta U - T\Delta S$ to extract the enthalpic and entropic contributions. The close agreement between the two methods highlights the accuracy of the MLP in describing the dissociation process.

single-wall carbon nanotubes (SWCNTs) of varying radii using the following expression (*87*):

$$E_{\mathrm{atom}}^{\mathrm{CNT}} = E_0 + S_0 B_M r^{-2}/2 \tag{S1}$$

where $E_{\mathrm{atom}}^{\mathrm{CNT}}$ is the energy per atom in a SWCNT, E_0 is the energy per atom in a flat graphene, and $S_0 = 2.63$ Å^2 is the planar footprint of a carbon atom in graphene.

By computing $E_{\mathrm{atom}}^{\mathrm{CNT}}$ for nanotubes with different radii, B_M can be obtained from the curvature dependence. As shown in Figure S6, our MLP accurately reproduces this curvature dependence, in excellent agreement with the reference *ab initio* values.

A similar procedure is applied to hBN, where the bending rigidity is obtained from boron nitride nanotubes (BNNT) with varying curvature, yielding excellent agreement with the reference data again, as shown in Figure S7.

Overall, the validations presented in this section demonstrate that the MLP developed in this work reliably captures the key physicochemical features governing water self-dissociation. From energetic and force accuracy across a range of conditions to its ability to reproduce temperature-dependent thermodynamic quantities and structural properties of graphene, the MLP provides a robust and transferable framework for investigating confined water reactivity with first-principles fidelity.

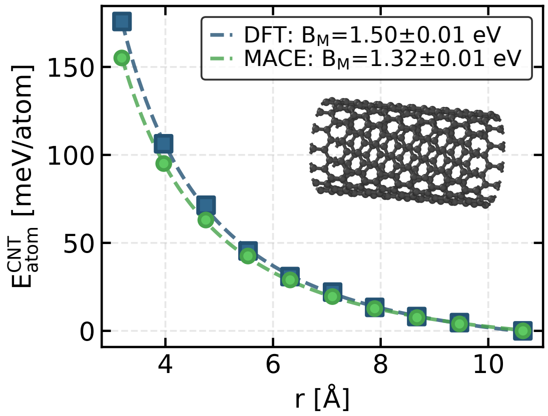

Figure S6: **Energy per atom as a function of nanotube radius for SWCNTs rolled along zigzag directions.** The dashed line indicates the fit used to extract the bending rigidity, B_M, of graphene. A representative SWCNT structure is shown as an inset.

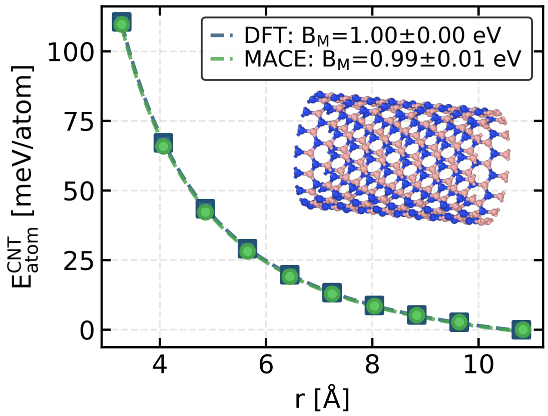

Figure S7: **Energy per atom as a function of nanotube radius for BNNTs rolled along zigzag directions.** The dashed line indicates the fit used to extract the bending rigidity, B_M, of hBN. A representative BNNT structure is shown as an inset.

3 Umbrella sampling

To investigate how water self-dissociation is affected by confinement, we performed 1D umbrella sampling simulations using a reaction coordinate defined as the coordination number of a selected oxygen atom (O^*) with all hydrogen atoms in the system:

$$n_H = \sum_{i=1}^{N} \frac{1 - (r_i/R_0)^{12}}{1 - (r_i/R_0)^{24}} \quad \text{(S2)}$$

where i iterates over all the hydrogens in the simulation box, r_i is the distance between hydrogen i and O^*, and R_0 =1.38 Å (*40*).

To sample configurations across the reaction coordinate, we applied a harmonic restraint around target values n'_H using a force constant of 200 kcal/mol per coordination unit squared. Each umbrella window was centered between n'_H = 1.00 and 2.20 in steps of 0.04, for a total of 31 windows per system. Each window was sampled for 100 ps.

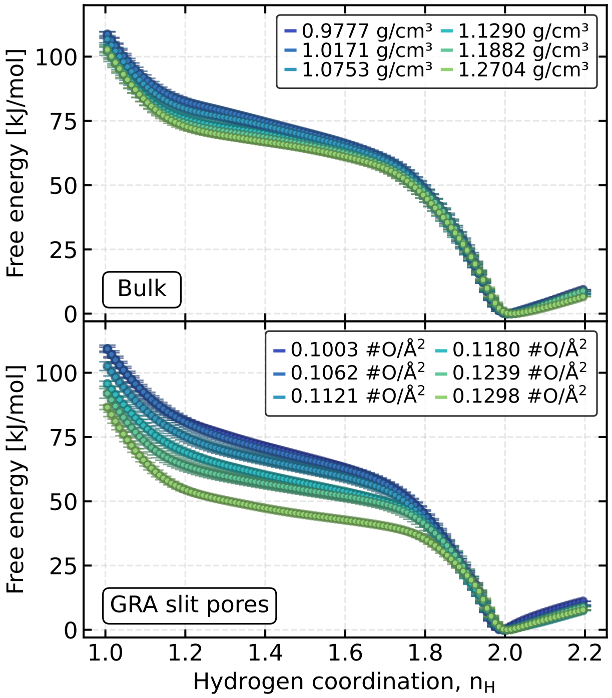

Figure S8: **Free energy profiles as a function of the hydrogen coordination number, n_H, for the H_2O self-dissociation reaction across the different systems, obtained using umbrella sampling.**

The resulting free energy profiles were reconstructed using umbrella integration (*77, 88*) (see Figure S8). The self-dissociation constant of water, $p\mathrm{K_w}$, was then computed from the free energy difference $\Delta F^{\ddagger}$ between $n_\mathrm{H} \approx 2.0$ and $n_\mathrm{H} \approx 1.2$ using the following expression:

$$p\mathrm{K_w} = \frac{\Delta F^{\ddagger}}{RT\ln(10)} \tag{S3}$$

where R is the molar gas constant, and T is the temperature.

Because our goal is to understand how confinement modulates water dissociation, we emphasize relative differences in $p\mathrm{K_w}$ across systems rather than precise absolute values. This strategy ensures that our conclusions remain robust and transferable, as they are less sensitive to the choice of electronic structure method, neglect of nuclear quantum effects, or the specific sampling protocol. While more complex approaches—such as multidimensional reaction coordinates—may offer increased absolute accuracy (*8, 9*), our systematic comparisons offer a reliable and meaningful picture of how confinement alters self-dissociation. Finally, we verified that finite-size effects do not impact the reported trends.

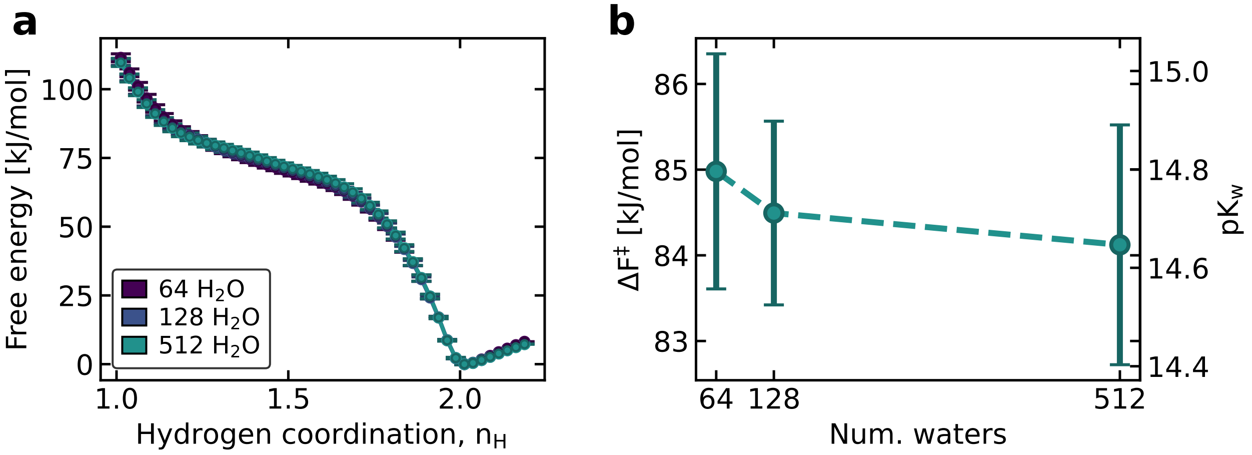

Figure S9: **Assessment of finite-size effects in water self-dissociation.** (a) Free energy profiles for the water self-dissociation reaction across systems with an increasing number of water molecules. (b) Finite size dependence of the computed free energy barrier $\Delta F^{\ddagger}$ (obtained as the free energy difference between $n_\mathrm{H} \approx 2$ an $n_\mathrm{H} \approx 1.2$) and self-dissociation constant of water $p\mathrm{K_w}$. Convergence in these values is achieved for systems exceeding 128 water molecules per unit cell.

4 Sensitivity of water self-dissociation to confinement width and pore flexibility

Figure S10 extends the analysis of water self-dissociation to different confinement widths, comparing monolayer (1L), bilayer (2L), and trilayer (3L) water confined in rigid graphene slit pores. Because selecting a unique equilibrium water content across different pore widths is inherently non-trivial, these systems are constructed at fixed two-dimensional surface density, with the bilayer and trilayer pores containing approximately two and three times the monolayer loading, respectively. Under these conditions, the dependence of $p\mathrm{K_w}$ on the number of layers in the rigid case can appear non-monotonic and sensitive to the specific choice of confinement and loading.

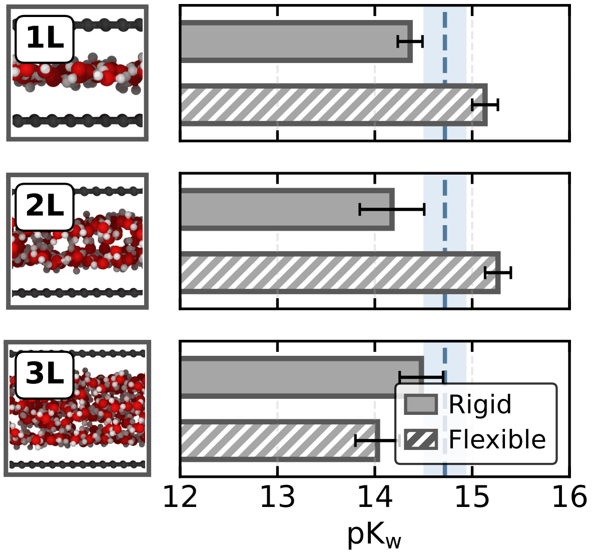

Figure S10: $p\mathrm{K_w}$ for 1L, 2L, and 3L graphene slit pores, showing the influence of pore width and flexibility on dissociation behavior under confinement. The rigid 1L, 2L, and 3L setups correspond to slit widths of 6.70, 10.05, and 13.40 Å, respectively. These values are commensurate with pore sizes that can be experimentally realized using van der Waals (vdW) assembly (*13,83*) . The vertical dashed blue line indicates our bulk reference estimate at 1 bar with its errors.

We further assess the role of pore flexibility. In contrast to rigid slit pores, flexible pores exhibit different trends, with thinner pores generally showing reduced dissociation. However, direct comparisons between rigid and flexible systems remain challenging, as flexible pores, even when

exhibiting a similar average interlayer spacing, can accommodate a wider range of densities and layering motifs (*42*). The situation is further complicated by distinct phase behavior. For example, bilayer water has been shown to exhibit ice-like structural characteristics, driven by an anomalously high melting temperature that exceeds that of bulk ice (*89, 90*).

5 Consistent thermodynamic comparison between bulk and confined water

To consistently compare how water self-dissociation changes with thermodynamic conditions in bulk and confined environments, we computed variations in the chemical potential. This approach is necessary as other thermodynamic variables, such as pressure, are ill-defined in nanoconfined systems due to the ambiguity in specifying an effective volume within the slit pore.

To quantify chemical potential changes under varying thermodynamic conditions, we first need to establish a reference chemical potential. For bulk water, this reference corresponds to the chemical potential of a 1 bar liquid water box, a standard condition widely used in both experiments and simulations to represent standard ambient liquid water. For nanoconfined water, the reference corresponds to the chemical potential at which the surface density within the central region of the slit pore reaches its equilibrium value, determined by extrapolating simulations across different system sizes. To this end, we simulated systems with two parallel graphene layers of equal length, periodic along the y-axis, lateral dimensions of 44.460 × 47.058 Å^2, 54.340 × 55.614 Å^2, 64.220 × 64.170 Å^2, and 74.100 × 77.004 Å^2, all immersed in liquid water (see Section S1 for details). For each system, we computed the oxygen surface density within the central region of the slit pore and extrapolated these results to the $L \rightarrow \infty$ limit to obtain the equilibrium density within the confined region, as shown in Figure S11.

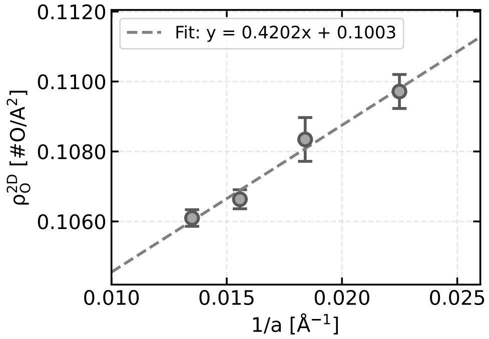

Figure S11: **Surface density as a function of $1/a$, where a is the graphene lattice constant along the x-axis (see Section S1).** The extrapolated density value for $L \rightarrow \infty$ limit corresponds to 0.1003 #O/Å^2.

Having established consistent reference chemical potentials, we can now directly compare the behavior of bulk and nanoconfined water under varying thermodynamic conditions. For large, homogeneous systems where the Gibbs free energy G scales linearly with the number of moles N, the chemical potential simplifies to:

$$\mu = \frac{G}{N} = G_m,$$

where G_m denotes the molar Gibbs free energy. Similarly, the molar entropy and molar volume are given by:

$$S_m = \frac{S}{N} \quad \text{and} \quad V_m = \frac{V}{N}.$$

The differential form of the Gibbs free energy is:

$$dG = -S\,dT + V\,dP,$$

which, expressed per mole, becomes:

$$d\mu = -S_m\,dT + V_m\,dP.$$

Since our simulations are performed at constant temperature, this simplifies to:

$$d\mu = V_m\,dP.$$

Integrating, we obtain:

$$\mu(P') - \mu^0(P_0) = \int_{P_0}^{P'} V_m\,dP, \tag{S4}$$

where $\mu^0(P_0)$ is the reference chemical potential. For bulk water, we evaluate Eq. S4 using $P_0 =$ 1 bar as the reference state.

In nanoconfined systems, however, as discussed earlier, specifying an effective confined volume introduces ambiguity, making direct application of Eq. S4 problematic. To overcome this, we reformulate Eq. S4 in a way that avoids the explicit definition of a confined volume. Starting from

$$\mu(P') - \mu^0(P_0) = \int_{P_0}^{P'} \frac{V}{N}\,dP,$$

we introduce the scalar total stress of the simulation cell, defined as $\sigma \equiv PV$, where P is the pressure obtained from the trace of the virial stress tensor and V is the total simulation-box volume. Here, the virial pressure is evaluated from the forces acting on the water molecules only; the confining carbon

atoms are held rigid and are therefore excluded. Crucially, while the individual numerical values of P and V depend on the chosen volume convention, their product σ is uniquely defined within the simulation. With this definition, variations in stress at constant volume satisfy $d\sigma = V\, dP$. The chemical potential difference can then be expressed as

$$\mu(\sigma') - \mu^0(\sigma_0) = \int_{\sigma_0}^{\sigma'} \frac{1}{N}\, d\sigma. \tag{S5}$$

In practice, the integral in Eq. S5 is evaluated using simulation data obtained at different water loadings. At each state point, the pressure is computed from the water contributions to the virial and multiplied by the total simulation-box volume, with both quantities taken as time-averaged values. By varying the number of confined water molecules, the equilibrium value of σ is changed, allowing the integral over $d\sigma$ to be constructed without defining an explicit confined volume or pressure.

Finally, to enable a consistent reference between bulk and nanoconfined water, we note that, as a direct consequence of our equilibration procedure, bulk and nanoconfined states can be related through equivalent thermodynamic reference conditions via

$$\mu^0_{\text{bulk}}(1\text{ bar}) \equiv \mu^0_{\text{conf}}(\rho^{\text{2D}}_{\text{O, eq.}}),$$

where $\rho^{\text{2D}}_{\text{O, eq.}}$ denotes the equilibrium surface density of confined water. With this choice, variations in $p\text{K}_\text{w}$ can be compared on a common thermodynamic footing between bulk and nanoconfined environments.

6 Determination of equilibrium density in nanodroplet confinement

To ensure a meaningful comparison with bulk water, we designed the nanodroplet-confined systems such that the droplet core reaches its equilibrium density–defined as the density at which structural properties within the droplet's interior converge with respect to system size. We then compared this state point to bulk water at its equilibrium density, thereby isolating the intrinsic effects of confinement without introducing artifacts from overpressurization.

In general, determining the equilibrium density in confinement is a nontrivial task. The literature presents a variety of approaches—including piston-based equilibration, flexible boundary conditions, or matching the average pore density to bulk experimental values—but these methods often yield widely varying results. Table S8 provides a selection of representative examples, including both force field and first-principles molecular dynamics studies, illustrating the range of reported densities for systems mostly under graphene confinement. This list is by no means comprehensive, as many additional studies exist. Nevertheless, the variation in reported values highlights the challenge of comparing water self-dissociation across studies, particularly given that the dissociation constant of water is known to decrease with increasing pressure.

Ref.	Method	n_{H_2O}	C–C distance [Å]	L_x [Å]	L_y [Å]	ρ_O^{2D} [#O/Å^2]
64	revPBE-D3	28	6.56	17.29	17.112	0.0946
91	revPBE-D3	16	6.63	12.30	12.78	0.1018
91	SPC/E	147	6.63	38.34	36.89	0.1039
83	revPBE-D3	248	6.70	44.46	47.058	0.1185
92	SPC/E	108	6.68	34.7484	34.3920	0.0904
93	SPC/E	27	6.63	17.12	17.30	0.0912
94	revPBE-D3	27	6.91	17.12	17.30	0.0912
95	PBE	38	6.98	19.71	21.37	0.09021

Table S8: **Monolayer water densities used in simulations of nanoslit-confined systems reported in various studies.** Here, n_{H_2O} denotes the number of water molecules, L_x and L_y refer to the graphene sheet dimensions in the x- and y-directions, respectively, and ρ_O^{2D} represents the two-dimensional surface density, defined as the number of oxygen atoms per unit area (O atoms/Å^2).

To accurately determine the equilibrium density in nanodroplet confinement, w e simulated a series of nanodroplets with varying dimensions and monitored the convergence of structural properties in the droplet core. To reduce the computational cost of these large-scale simulations, we first employed classical force field methods using the i-PI program (*84*) connected to the LAMMPS package (*80*). Water–water interactions were modeled using the TIP4P potential and water–carbon interactions with the AIREBO potential (*96*).

We initially simulated a large nanodroplet system with dimensions 98.800 Å × 98.394 Å to identify the density at which structural properties converge, as detailed in Table S9. The equilibrium density was determined by computing the cumulative number of oxygen atoms, $N_O(r)$, as a function of radial distance r from the droplet center. The 2D oxygen density, $\rho_O^{2D}(r)$, was then obtained from the average slope of multiple linear fits to this curve:

$$\rho_O^{2D}(r) = \frac{1}{2\pi r}\frac{dN_O(r)}{dr}. \quad \text{(S6)}$$

This method provides a smooth, binning-free estimate of the density, minimizing artifacts from interfacial fluctuations and statistical noise.

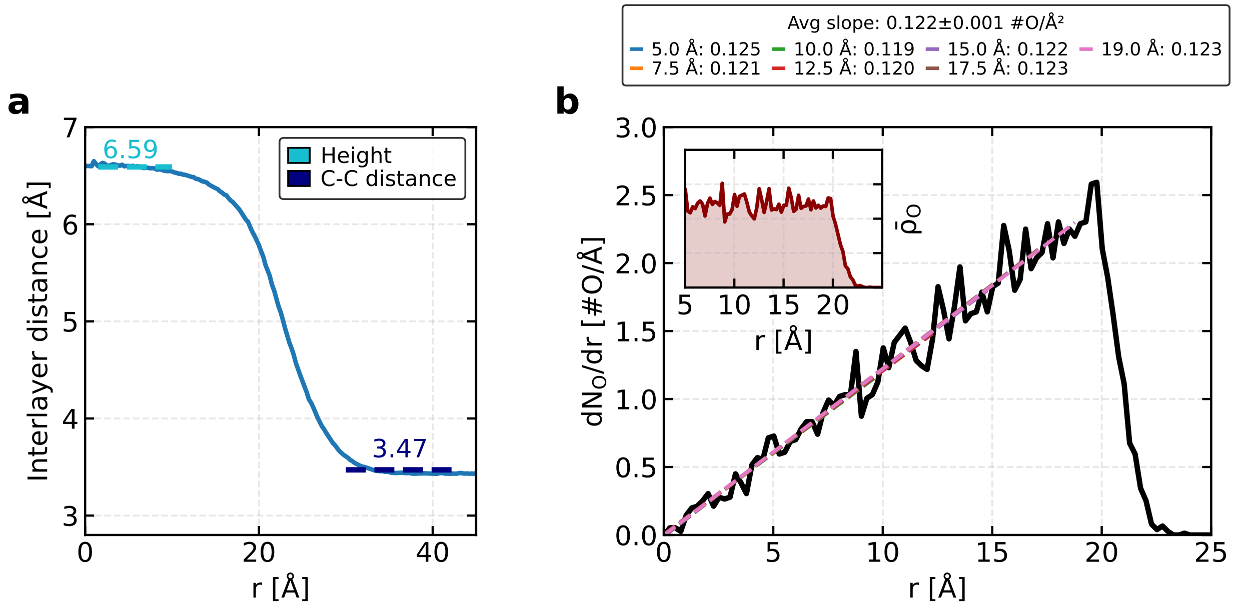

Figure S12: **Nanodroplet equilibration and characterization using a classical force field.** (a) Interlayer distance as a function of the nanodroplet radius, obtained using the classical force field described. The droplet height is determined from the central region, where water molecules are present, while the C–C distance is computed from the outer region, where water is absent. (b) Cumulative number of oxygen atoms as a function of the nanodroplet radius, obtained using the classical force field described. The 2D oxygen density, ρ_O^{2D}, is determined from the average slope of multiple linear fits. Each fit region and its corresponding slope are reported in the legend. The inset shows the normalized radial density profile of oxygen atoms, $\bar{\rho}_O$.

Following this procedure, we determined the equilibrium density for a system with sheet dimensions of 98.800 Å × 98.394 Å. As shown in Table S9, convergence of the core structural properties is achieved when 132 water molecules are confined within the droplet.

Sheet dimensions (Å^2)	n_{H_2O}	Droplet height [Å]	C–C distance [Å]	Droplet radius [Å]	ρ_O^{2D} [#O/Å^2]
98.800 × 98.394	33	6.31	3.43	11.1	0.110 ± 0.010
98.800 × 98.394	66	6.46	3.44	14.9	0.122 ± 0.010
98.800 × 98.394	99	6.55	3.43	17.9	0.124 ± 0.005
98.800 × 98.394	132	6.61	3.43	20.4	0.120 ± 0.006
98.800 × 98.394	165	6.64	3.45	22.71	0.120 ± 0.002
98.800 × 98.394	198	6.66	3.43	24.74	0.121 ± 0.002
98.800 × 98.394	231	6.65	3.44	26.59	0.122 ± 0.002

Table S9: **Convergence of structural properties for a system with dimensions of 98.800 Å × 98.394 Å^2, evaluated as a function of the number of water molecules n_{H_2O}.** Core structural features converge when 132 water molecules are present within the droplet.

Since our goal is to ultimately simulate this nanodroplet using the MACE MLP developed in this work, which is significantly more computationally demanding than classical force fields, we investigated whether the system size could be reduced without compromising equilibrium properties. By fixing the number of water molecules at 132 and varying the graphene sheet dimensions, we found that a system size of 79.040 Å × 81.282 Å is sufficient to preserve the target density in the droplet core (see Table S10).

Sheet dimensions (Å^2)	n_{H_2O}	Droplet height [Å]	C–C distance [Å]	Droplet radius [Å]	ρ_O^{2D} [#O/Å^2]
59.280 × 59.892	132	6.58	3.46	20.5	0.121 ± 0.002
79.040 × 81.282	132	6.61	3.44	20.5	0.120 ± 0.002
98.800 × 98.394	132	6.61	3.43	20.4	0.120 ± 0.006

Table S10: **Convergence of structural properties for a system with 132 water molecules, evaluated as a function of the sheet dimensions.** Core structural features converge with sheet dimensions of 79.040 Å × 81.282 Å.

Based on these results, the nanodroplet system used for simulations with the MLP developed in this work consisted of 132 water molecules confined between graphene sheets with dimensions of 79.040 Å × 81.282 Å. To better reflect experimental conditions, the bottom graphene sheet was held fixed, mimicking graphene on a substrate, covered by an additional flexible graphene sheet. Following the same procedure described earlier, we determined the equilibrium density for this system using the MLP. As we see in Figure S13, the value obtained corresponds to ρ_O^{2D} = 0.118 #O/Å^2, which is the one reported in the main part of the manuscript and in Section 1.

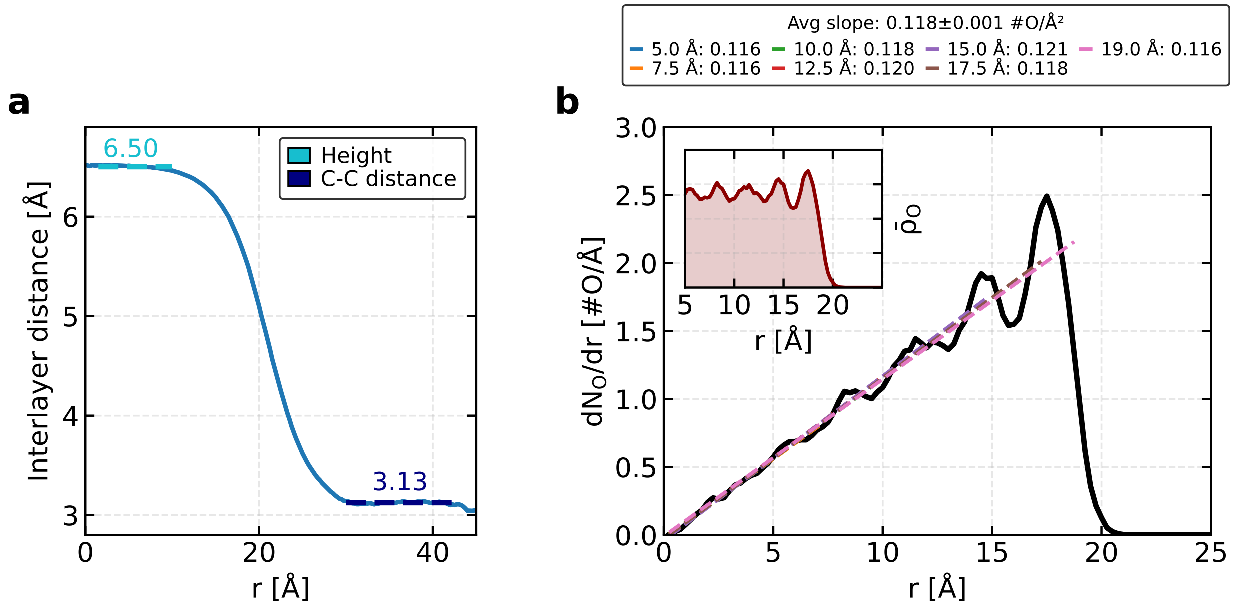

Figure S13: **Nanodroplet equilibration and characterization using the MLP developed in this work.** (a) Interlayer distance as a function of the nanodroplet radius, obtained using the MLP developed in this work. The droplet height is determined from the central region, where water molecules are present, while the C–C distance is computed from the outer region, where water is absent. (b) Cumulative number of oxygen atoms as a function of the nanodroplet radius, obtained using the MLP developed in this work. The 2D oxygen density, ρ_O^{2D}, is determined from the average slope of multiple linear fits. Each fit region and its corresponding slope are reported in the legend. The inset shows the normalized radial density profile of oxygen atoms, $\bar{\rho}_O$.

7 Water self-dissociation in bulk and nanodroplet-confined systems

The pKw values for water confined within graphene and hBN nanodroplets are obtained from the free energy profiles of the self-dissociation reaction shown in Figure S14, using the relationship defined in Equation S3.

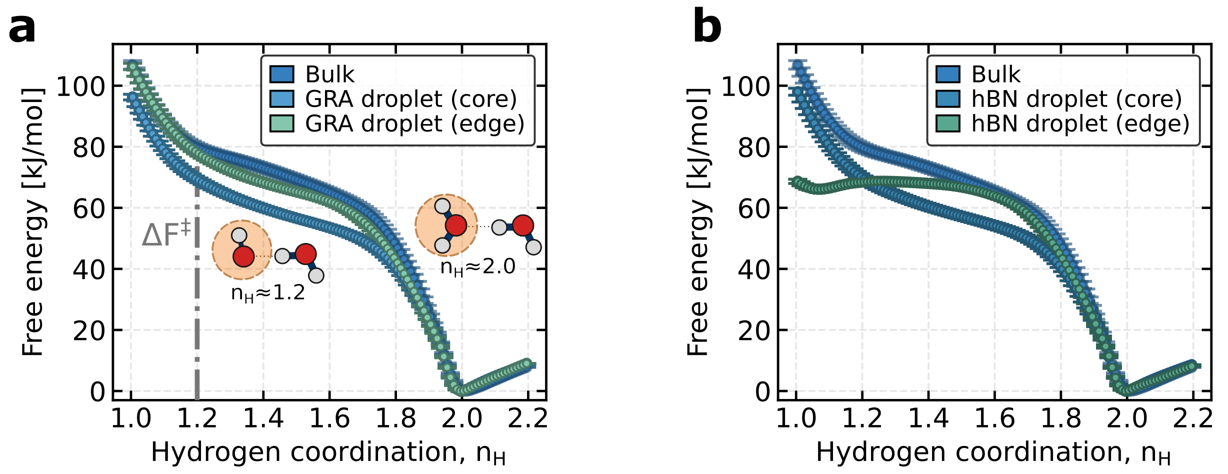

Figure S14: **Free energy profiles for the water self-dissociation reaction across the different systems.** The free energy barrier $\Delta F^{\ddagger}$, used to compute pKw, is defined as the free energy difference between $n_{\mathrm{H}} \approx 2$ and $n_{\mathrm{H}} \approx 1.2$. For dissociation events occurring at the edge of the hBN nanodroplet, we instead use $n_{\mathrm{H}} \approx 1.05$.

To characterize structural variations relevant to water self-dissociation, we analyzed the O–O separations in O–H···O pairs as a function of their location within the nanodroplet. For each such pair, we introduced a radial coordinate $\tilde{r}$ defined as the distance between the droplet center of mass (computed from the oxygen atoms) and the midpoint of the corresponding O–O vector, which represents the center of the hydrogen-bonded pair. Based on this coordinate, pairs were classified as belonging to the "core" or "edge" regions depending on whether $\tilde{r}$ lies below or above a chosen cutoff value (see Figure S15a). Figure S15b illustrates how the O–O distances in O–H···O pairs vary with the radial cutoff $\tilde{r}_{\text{cut}}$. We find that a value of $\tilde{r}_{\text{cut}} \approx 14.5$ Å provides a distinction between core- and edge-associated pairs, capturing the characteristic structural differences between the two regions. This choice is further justified by the analysis presented later in this section, where several observables exhibit their most pronounced contrast between core and edge regions near $\tilde{r}_{\text{cut}} \approx 14.5$ Å. Accordingly, Figure 4 in the main manuscript reports representative O–O distances for the core and edge regions using $\tilde{r} = 14.5$ Å as the boundary between the two.

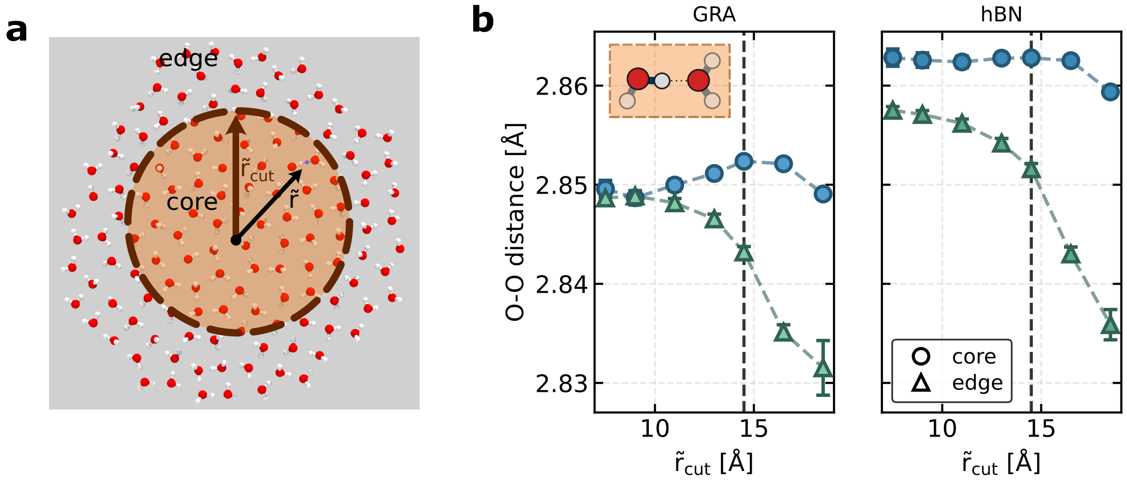

Figure S15: **Core–edge definition and O–O distances in O–H···O pair in water nanodroplets.** (a) Schematic illustration of the core–edge classification within a nanodroplet. For each O–H···O pair, the radial coordinate $\tilde{r}$ is defined as the distance between the droplet center of mass (oxygen atoms) and the midpoint of the O–O vector. Pairs with $\tilde{r} < \tilde{r}_{\text{cut}}$ are labeled "core" and those with $\tilde{r} \geq \tilde{r}_{\text{cut}}$ are labeled "edge". (b) O–O distances in O–H···O pairs as a function of $\tilde{r}_{\text{cut}}$ for water confined in graphene and hBN nanodroplets. The accompanying schematic illustrates the definition of the O–H···O geometry used in the analysis.

In the main text, we compare the structural properties of water in the core and edge regions of graphene- and hBN-encapsulated nanodroplets to rationalize the emergence of distinct local solvation environments. Figures S16 and S17 illustrate the development of distinct core and edge regions in graphene- and hBN-encapsulated water nanodroplets, as well as the microscopic differences between the two systems. Panels (a) of Figs. S16 and S17 show the spatial variation of the interlayer distance across the confining sheets, revealing a core region surrounded by an edge region in which the confinement progressively softens. Panels (b–d) quantify how the local solvation environment of water evolves from the core to the edge of the nanodroplets. As a function of radial distance from the droplet center, we observe a pronounced reduction in the oxygen–oxygen coordination number, a concomitant decrease in the number of hydrogen bonds per molecule, including both donor and acceptor contributions, and changes in the average O–O distance within hydrogen-bonded O–H$\cdots$O pairs. While graphene and hBN exhibit different structural responses at the droplet edge, with hBN showing distinct hydrogen-bonded O–O distances due to the presence of a chemisorbed hydroxide species, both systems share the emergence of undercoordinated and weakly hydrogen-bonded interfacial water molecules.

Similar undercoordinated water environments are known to occur at the air–water interface, which we use in the main text as a reference point to illustrate the structural consequences of reduced coordination and disrupted hydrogen bonding at an interface. In the present confined nanodroplets, the core–edge variation in coordination and hydrogen-bond connectivity is expected to modify the local electrostatic screening experienced by water molecules and proton defects, thereby altering the free energy balance of water autoionization. This provides a direct and system-specific microscopic basis for the observed spatial variation of pKw within the nanodroplets, linking changes in autoionization thermodynamics to the local solvation environment at the interface.

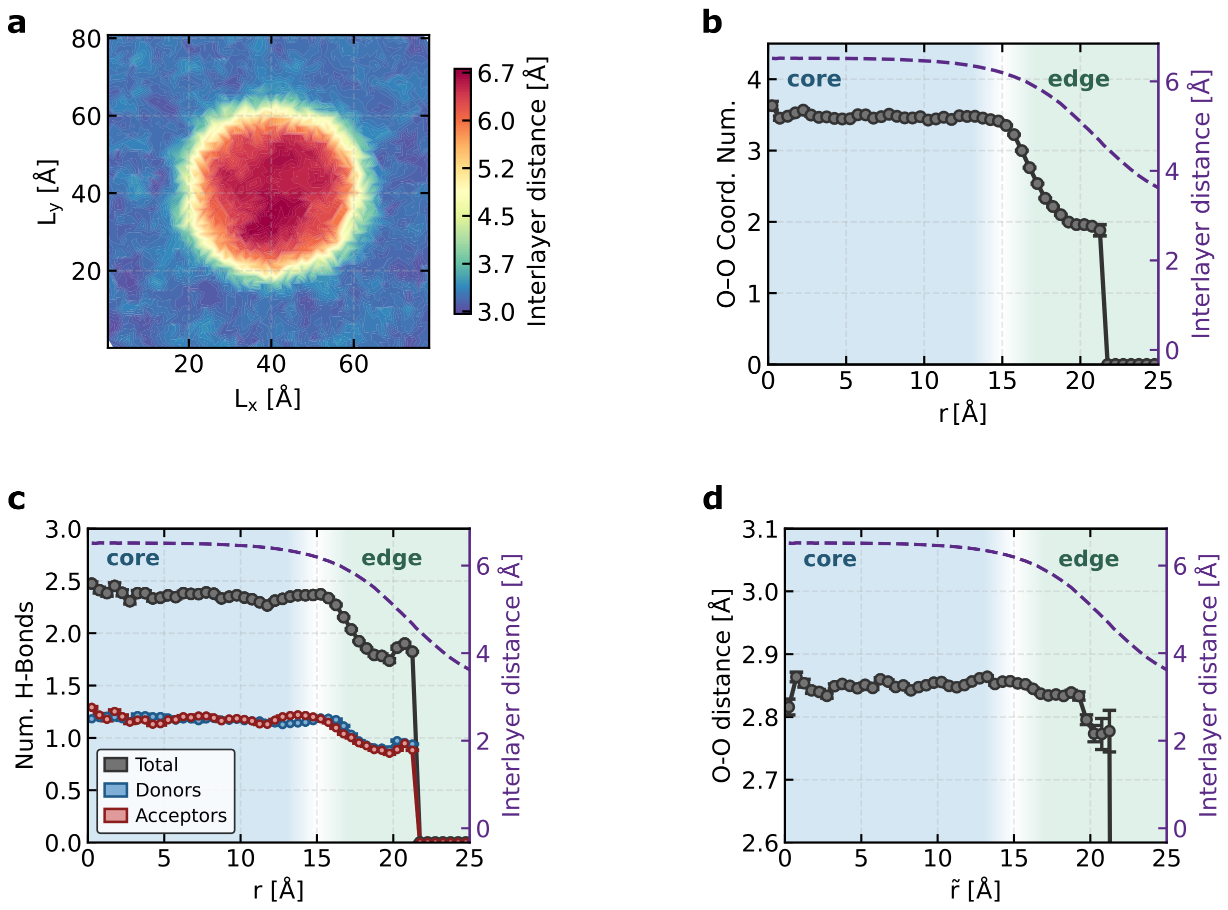

Figure S16: **Structural and hydrogen-bonding properties of water in graphene-encapsulated nanodroplets.** (a) Spatial map of the interlayer distance between the confining graphene sheets, showing the variation of the confinement across the nanodroplet and the emergence of distinct core and edge regions. (b) Oxygen–oxygen coordination number as a function of the radial distance from the droplet center, r, showing the change in local coordination between the core and edge regions. (c) Average number of hydrogen bonds per water molecule as a function of the radial distance from the droplet center, r. Total hydrogen bonds are shown, along with the separate contributions from donors and acceptors. (d) Average oxygen–oxygen distance within hydrogen-bonded O–H$\cdots$O pairs as a function of $\tilde{r}$, which denotes the distance between the droplet center of mass and the midpoint of the corresponding O–O vector defining each hydrogen-bonded pair. In panels (b–d), the faded blue and green backgrounds indicate the core and edge regions of the nanodroplet, respectively. The interlayer distance (droplet height) is superimposed on a secondary y-axis as a function of r. The identification of the core and edge regions follows the definition introduced in Figure S15, based on the radial variation of the droplet height.

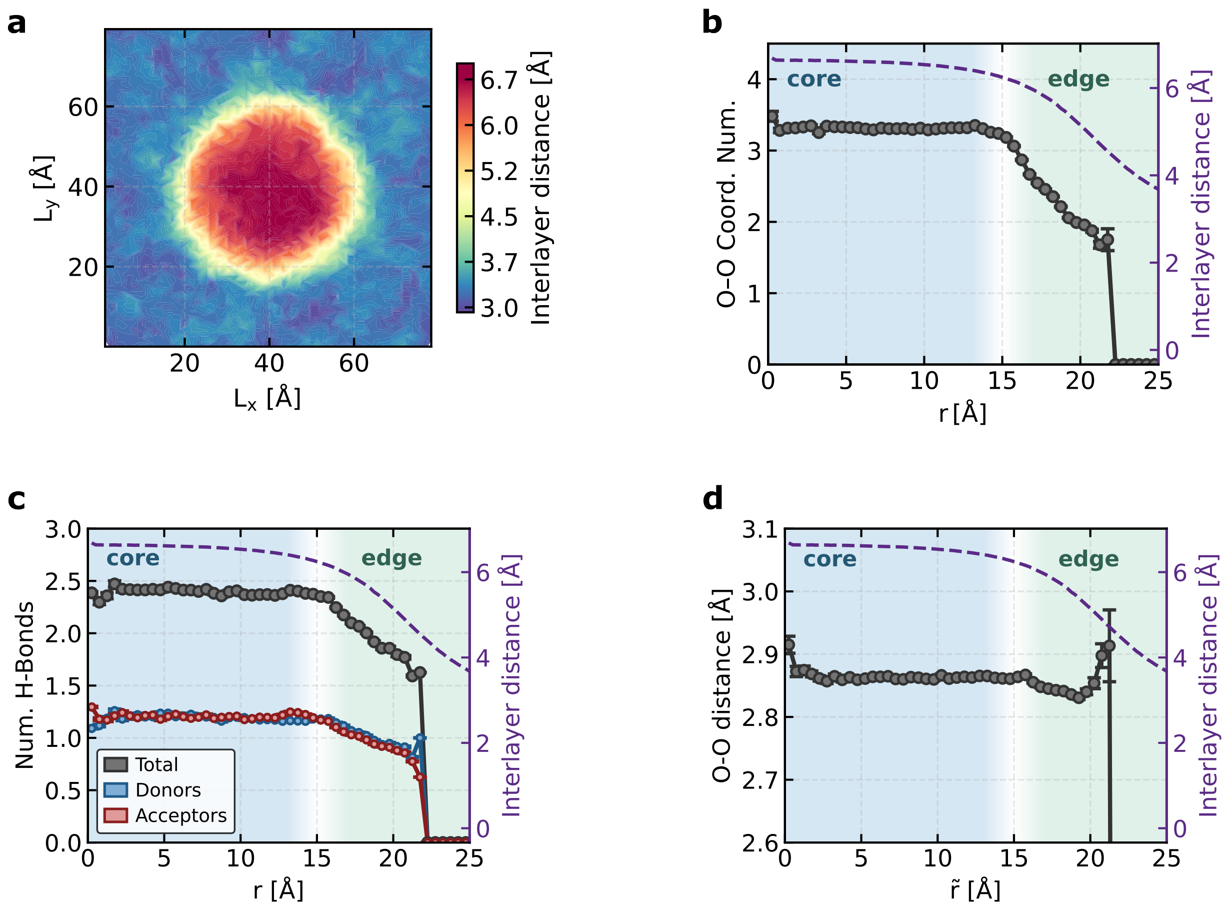

Figure S17: **Structural and hydrogen-bonding properties of water in hBN-encapsulated nanodroplets.** (a) Spatial map of the interlayer distance between the confining hBN sheets, showing the variation of the confinement across the nanodroplet and the emergence of distinct core and edge regions. (b) Oxygen–oxygen coordination number as a function of the radial distance from the droplet center, r, showing the change in local coordination between the core and edge regions. (c) Average number of hydrogen bonds per water molecule as a function of the radial distance from the droplet center, r. Total hydrogen bonds are shown, along with the separate contributions from donors and acceptors. (d) Average oxygen–oxygen distance within hydrogen-bonded O–H$\cdots$O pairs as a function of $\tilde{r}$, which denotes the distance between the droplet center of mass and the midpoint of the corresponding O–O vector defining each hydrogen-bonded pair. In panels (b–d), the faded blue and green backgrounds indicate the core and edge regions of the nanodroplet, respectively. The interlayer distance (droplet height) is superimposed on a secondary y-axis as a function of r. The identification of the core and edge regions follows the definition introduced in Figure S15, based on the radial variation of the droplet height.

8 Discussion in the Context of Previous Work

In the main text, we mention that our results clarify how the apparently conflicting findings reported in the literature can be reconciled, with titles including *"Nanoconfinement in Slit Pores Enhances Water Self-Dissociation"* (*32*), *"Water Self-Dissociation is Insensitive to Nanoscale Environments"* (*33*), and *"Sub-nanometer Confinement Suppresses Autoionization of Water"* (*34*). In particular, our study shows that, for water dissociation under confinement, the devil is in the details. Quantitative comparisons between different studies are highly dependent on how the thermodynamic reference state of the confined liquid is defined. Differences in equilibration protocols, water loading, interaction models, and interfacial stabilization mechanisms can place the confined water in distinct thermodynamic states, even when simulations are nominally performed at the same external pressure. Recognizing this extreme sensitivity was a central motivation for the systematic and thorough analysis presented in this work.

For a more detailed discussion, we summarize work in the literature in the table below, which facilitates comparison and helps resolve the apparent inconsistencies. Where available, we focus on results obtained under monolayer confinement, as this allows a more meaningful comparison of the underlying physical factors controlling water dissociation.

	Dasgupta *et al.* Ref. 34	Di Pino *et al.* Ref. 33	Di Pino *et al.* Ref. 33	Muñoz-Santiburcio & Marx Ref. 32	This work
Equilibration method	Piston (1 atm)	Ext. reservoir (finite-size uncorrected)	Ext. reservoir (finite-size uncorrected)	Expt. data (& sensitivity analysis)	Ext. reservoir (finite-size corrected)
Pore width [nm]	0.66	1.20	0.94	~1	0.67
$\rho_{\mathrm{O}}^{\mathrm{2D}}$ [num. O/Å^2]	~0.11	0.2757	0.212	~0.13	0.1003
Wall treatment	Implicit	Implicit	Implicit	Explicit	Explicit
Confinement regime	Monolayer	Trilayer	Bilayer	Bilayer	Monolayer
pK_{w}	> bulk	> bulk	> bulk	≈ bulk	≈ bulk

Table S11: **Comparison of equilibration protocols, confinement parameters, and resulting water dissociation behavior reported in the literature and in the present work.**

- **Comparison to Ref. 34.** While both Ref. 34 and the present work examine nominally monolayer-confined water, they employ different electronic-structure descriptions and confinement models. Ref. 34 uses density-corrected r^{2}SCAN, whereas the present work is based on revPBE-D3(0). These choices have consequences for both bulk water properties and the dissociation equilibrium, as r^{2}SCAN typically requires explicit nuclear quantum effects to reproduce a realistic bulk $p\mathrm{K_w}$, while revPBE-D3(0) yields a reasonable bulk reference due to a fortuitous cancellation of errors (*97*). In addition, the two studies differ in their treatment of water–surface interactions: Ref. 34 employs effective wall–water models, whereas here the confining surfaces (graphene and hBN) are treated explicitly at the electronic-structure level. This distinction is important since, as noted in Ref. 34, variations in wall–water interactions modulate the hydrogen-bond network and shift the ionization equilibrium. Finally, the con-fined water density in Ref. 34 is set using a piston-based approach designed to stabilize a monolayer at nominally 1 atm. While this defines a clear confinement protocol, it does not uniquely determine the surface density of the confined liquid, as nominal monolayers are known to span a range of surface densities (see Figure 1 in Ref. 42).

- **Comparison to Ref. 33.** In contrast to the present work, which focuses on monolayer confinement, Ref. 33 primarily considers bilayer- and trilayer-confined water. Moreover, Ref. 33 employs a QM/MM framework, introducing differences in both water–water and water–surface interactions relative to the first-principles level description used here. Importantly, the authors explicitly note that water filling in hydrophobic pores depends sensitively on surface hydrophobicity, implying that differences in contact angle directly translate into differences in the equilibrium confined water density. Using our revPBE-D3(0) model, we obtain a graphene–water contact angle of $72.1 \pm 1.5°$ (*98*), indicating a more moderately hydrophobic graphene–water interface than that in Ref. 33. This would tend to bring the confined water closer to bulk-like conditions and reduce deviations in $p\mathrm{K_w}$ (see Figure S3 in Ref. 33). Moreover, reservoir-equilibrated setups can be sensitive to finite-size effects associated with particle exchange. In the present work, we explicitly address this issue by demonstrating convergence through systematic system-size scaling and finite-size corrections. By contrast, the setup employed in Ref. 33, with dimensions of 70.0 Å × 29.8 Å × 80.0 Å, is likely to be

susceptible to finite-size effects, as evident from the scaling simulations in our study. We also note that bilayer-confined systems are known to exhibit particularly complex structural behavior, which can strongly influence both structural and reactive properties (*59, 90*).

- **Comparison to Ref. 32.** This study is closest to the present work in terms of electronic-structure description, but differs in the nature of the confining environment. In particular, in Ref. 32 they consider bilayer-confined water within a chemically distinct confining environment (mackinewite sheets) compared to the monolayer graphene and hBN systems considered here. Beyond the distinction between bilayer and monolayer confinement, the choice of confining material introduces substantially different water–surface interaction strengths, which can modify both the effective thermodynamic conditions experienced by the confined liquid and the stabilization of protonic defects (*60, 61*).

  In Ref. 32, the confined water content is determined by extrapolating experimental data rather than by explicit equilibration with a bulk reservoir, although extensive sensitivity analyses are performed. The observation of bulk-like $p\mathrm{K_w}$ values at effective pressures in the gigapascal range is therefore not inconsistent with our findings, but rather underscores that pressure alone does not uniquely determine dissociation behavior under confinement; material-specific interactions and solvation structure play a central role.

Overall, the comparisons above show that direct comparisons across studies are intrinsically challenging and highly sensitive to methodological details, and that differences in reported dissociation behavior largely reflect variations in confinement regime, equilibration protocol, and wall–water interactions, rather than confinement alone.

REFERENCES

1. P. L. Geissler, C. Dellago, D. Chandler, J. Hutter, M. Parrinello, Autoionization in liquid water. *Science* **291**, 2121–2124 (2001).

2. S. Z. Oener, M. J. Foster, S. W. Boettcher, Accelerating water dissociation in bipolar membranes and for electrocatalysis. *Science* **369**, 1099–1103 (2020).

3. P. Li, Y. Jiang, Y. Hu, Y. Men, Y. Liu, W. Cai, S. Chen, Hydrogen bond network connectivity in the electric double layer dominates the kinetic pH effect in hydrogen electrocatalysis on Pt. *Nat. Catal.* **5**, 900–911 (2022).

4. X. Chen, J. Chen, H. Chen, Q. Zhang, J. Li, J. Cui, Y. Sun, D. Wang, J. Ye, L. Liu, Promoting water dissociation for efficient solar driven CO_2 electroreduction via improving hydroxyl adsorption. *Nat. Commun.* **14**, 751 (2023).

5. A. Hassanali, M. K. Prakash, H. Eshet, M. Parrinello, On the recombination of hydronium and hydroxide ions in water. *Proc. Natl. Acad. Sci. U.S.A.* **108**, 20410–20415 (2011).

6. N. Agmon, H. J. Bakker, R. K. Campen, R. H. Henchman, P. Pohl, S. Roke, M. Thämer, A. Hassanali, Protons and hydroxide ions in aqueous systems. *Chem. Rev.* **116**, 7642–7672 (2016).

7. M. Moqadam, A. Lervik, E. Riccardi, V. Venkatraman, B. K. Alsberg, T. S. van Erp, Local initiation conditions for water autoionization. *Proc. Natl. Acad. Sci. U.S.A.* **115**, E4569–E4576 (2018).

8. T. Joutsuka, Molecular mechanism of autodissociation in liquid water: Ab initio molecular dynamics simulations. *J. Phys. Chem. B.* **126**, 4565–4571 (2022).

9. M. C. Andrade, R. Car, A. Selloni, Probing the self-ionization of liquid water with ab initio deep potential molecular dynamics. *Proc. Natl. Acad. Sci. U.S.A.* **120**, e2302468120 (2023).

10. G. Algara-Siller, O. Lehtinen, F. C. Wang, R. R. Nair, U. Kaiser, H. A. Wu, A. K. Geim, I. V. Grigorieva, Square ice in graphene nanocapillaries. *Nature* **519**, 443–445 (2015).

11. V. Kapil, C. Schran, A. Zen, J. Chen, C. J. Pickard, A. Michaelides, The first-principles phase diagram of monolayer nanoconfined water. *Nature* **609**, 512–516 (2022).

12. P. Ravindra, X. R. Advincula, C. Schran, A. Michaelides, V. Kapil, Quasi-one-dimensional hydrogen bonding in nanoconfined ice. *Nat. Commun.* **15**, 7301 (2024).

13. B. Radha, A. Esfandiar, F. C. Wang, A. P. Rooney, K. Gopinadhan, A. Keerthi, A. Mishchenko, A. Janardanan, P. Blake, L. Fumagalli, M. Lozada-Hidalgo, S. Garaj, S. J. Haigh, I. V. Grigorieva, H. A. Wu, A. K. Geim, Molecular transport through capillaries made with atomic-scale precision. *Nature* **538**, 222–225 (2016).

14. H. Yoshida, V. Kaiser, B. Rotenberg, L. Bocquet, Dripplons as localized and superfast ripples of water confined between graphene sheets. *Nat. Commun.* **9**, 1496 (2018).

15. P. Robin, T. Emmerich, A. Ismail, A. Niguès, Y. You, G. H. Nam, A. Keerthi, A. Siria, A. K. Geim, B. Radha, L. Bocquet, Long-term memory and synapse-like dynamics in two-dimensional nanofluidic channels. *Science* **379**, 161–167 (2023).

16. J. Jiang, Y. Gao, L. Li, Y. Liu, W. Zhu, C. Zhu, J. S. Francisco, X. C. Zeng, Rich proton dynamics and phase behaviours of nanoconfined ices. *Nat. Phys.* **20**, 456–464 (2024).

17. L. Fumagalli, A. Esfandiar, R. Fabregas, S. Hu, P. Ares, A. Janardanan, Q. Yang, B. Radha, T. Taniguchi, K. Watanabe, G. Gomila, K. S. Novoselov, A. K. Geim, Anomalously low dielectric constant of confined water. *Science* **360**, 1339–1342 (2018).

18. R. Wang, M. Souilamas, A. Esfandiar, R. Fabregas, S. Benaglia, H. Nevison-Andrews, Q. Yang, J. Normansell, P. Ares, G. Ferrari, A. Principi, A. K. Geim, L. Fumagalli, In-plane dielectric constant and conductivity of confined water. *Nature* **646**, 606–610 (2025).

19. T. Dwars, E. Paetzold, G. Oehme, Reactions in micellar systems. *Angew. Chem. Int. Ed. Engl.* **44**, 7174–7199 (2005).

20. D. Muñoz-Santiburcio, D. Marx, Chemistry in nanoconfined water. *Chem. Sci.* **8**, 3444–3452 (2017).

21. A. B. Grommet, M. Feller, R. Klajn, Chemical reactivity under nanoconfinement. *Nat. Nanotechnol.* **15**, 256–271 (2020).

22. Z. Wei, Y. Li, R. G. Cooks, X. Yan, Accelerated reaction kinetics in microdroplets: Overview and recent developments. *Annu. Rev. Phys. Chem.* **71**, 31–51 (2020).

23. M. de la Puente, D. Laage, How the acidity of water droplets and films is controlled by the air-water interface. *J. Am. Chem. Soc.* **145**, 25186–25194 (2023).

24. D. Muñoz-Santiburcio, D. Marx, Confinement-controlled aqueous chemistry within nanometric slit pores. *Chem. Rev.* **121**, 6293–6320 (2021).

25. L. Bocquet, E. Charlaix, Nanofluidics, from bulk to interfaces. *Chem. Soc. Rev.* **39**, 1073–1095 (2010).

26. M. Lozada-Hidalgo, S. Zhang, S. Hu, V. G. Kravets, F. J. Rodriguez, A. Berdyugin, A. Grigorenko, A. K. Geim, Giant photoeffect in proton transport through graphene membranes. *Nat. Nanotechnol.* **13**, 300–303 (2018).

27. M. Jaugstetter, N. Blanc, M. Kratz, K. Tschulik, Electrochemistry under confinement. *Chem. Soc. Rev.* **51**, 2491–2543 (2022).

28. R. J. Gomes, R. Kumar, H. Fejzić, B. Sarkar, I. Roy, C. V. Amanchukwu, Modulating water hydrogen bonding within a non-aqueous environment controls its reactivity in electrochemical transformations. *Nat. Catal.* **7**, 689–701 (2024).

29. D. Deng, K. S. Novoselov, Q. Fu, N. Zheng, Z. Tian, X. Bao, Catalysis with two-dimensional materials and their heterostructures. *Nat. Nanotechnol.* **11**, 218–230 (2016).

30. J. Chen, X.-Z. Li, Q. Zhang, A. Michaelides, E. Wang, Nature of proton transport in a water-filled carbon nanotube and in liquid water. *Phys. Chem. Chem. Phys.* **15**, 6344–6349 (2013).

31. Y. A. P. Sirkin, A. Hassanali, D. A. Scherlis, One-dimensional confinement inhibits water dissociation in carbon nanotubes. *J. Phys. Chem. Lett.* **9**, 5029–5033 (2018).

32. D. Muñoz Santiburcio, D. Marx, Nanoconfinement in slit pores enhances water self-dissociation. *Phys. Rev. Lett.* **119**, 056002 (2017).

33. S. Di Pino, Y. A. P. Sirkin, U. N. Morzan, V. M. Sánchez, A. Hassanali, D. A. Scherlis, Water self-dissociation is insensitive to nanoscale environments. *Angew. Chem. Int. Ed. Engl.* **135**, e202306526 (2023).

34. S. Dasgupta, S. Saha, F. Paesani, Sub-nanometer confinement suppresses autoionization of water. *J. Am. Chem. Soc.* **147**, 25167–25173 (2025).

35. Y. Hashikawa, S. Hasegawa, Y. Murata, A single but hydrogen-bonded water molecule confined in an anisotropic subnanospace. *Chem. Commun.* **54**, 13686–13689 (2018).

36. M. J. Prieto, T. Mullan, M. Schlutow, D. M. Gottlob, L. C. Tănase, D. Menzel, J. Sauer, D. Usvyat, T. Schmidt, H. J. Freund, Insights into reaction kinetics in confined space: Real time observation of water formation under a silica cover. *J. Am. Chem. Soc.* **143**, 8780–8790 (2021).

37. A. Siria, P. Poncharal, A. L. Biance, R. Fulcrand, X. Blase, S. T. Purcell, L. Bocquet, Giant osmotic energy conversion measured in a single transmembrane boron nitride nanotube. *Nature* **494**, 455–458 (2013).

38. G. Tocci, L. Joly, A. Michaelides, Friction of water on graphene and hexagonal boron nitride from ab initio methods: Very different slippage despite very similar interface structures. *Nano Lett.* **14**, 6872–6877 (2014).

39. B. Grosjean, M.-L. Bocquet, R. Vuilleumier, Versatile electrification of two-dimensional nanomaterials in water. *Nat. Commun.* **10**, 1656 (2019).

40. M. Sprik, Computation of the pK of liquid water using coordination constraints. *Chem. Phys.* **258**, 139–150 (2000).

41. Y. Litman, A. Michaelides, Entropy governs the structure and reactivity of water dissociation under electric fields. *J. Am. Chem. Soc.* **147**, 44885–44894 (2025).

42. L. R. Pestana, L. E. Felberg, T. Head-Gordon, Coexistence of multilayered phases of confined water: The importance of flexible confining surfaces. *ACS Nano* **12**, 448–454 (2018).

43. A. V. Bandura, S. N. Lvov, The ionization constant of water over wide ranges of temperature and density. *J. Phys. Chem. Ref. Data Monogr.* **35**, 15–30 (2005).

44. S. Ruiz-Barragan, D. Muñoz-Santiburcio, S. Körning, D. Marx, Water self-dissociation in slit pores displays non-monotonic behavior as a function of water filling. *Chem. Sci.* **17**, 7447–7453 (2026).

45. A. Nitzan, *Chemical dynamics in condensed phases: Relaxation, transfer, and reactions in condensed molecular systems* (Oxford Univ. Press, 2024).

46. S. J. Cox, P. L. Geissler, Dielectric response of thin water films: A thermodynamic perspective. *Chem. Sci.* **13**, 9102–9111 (2022).

47. J. Zubeltzu, F. Bresme, M. Dawber, M. Fernandez-Serra, E. Artacho, Redefining the dielectric response of nanoconfined liquids: Insights from water. *Phys. Rev. Res.* **7**, 043101 (2025).

48. N. Severin, P. Lange, I. M. Sokolov, J. P. Rabe, Reversible dewetting of a molecularly thin fluid water film in a soft graphene–mica slit pore. *Nano Lett.* **12**, 774–779 (2012).

49. D. Lee, G. Ahn, S. Ryu, Two-dimensional water diffusion at a graphene-silica interface. *J. Am. Chem. Soc.* **136**, 6634–6642 (2014).

50. A. C. Forse, C. Merlet, J. M. Griffin, C. P. Grey, New perspectives on the charging mechanisms of supercapacitors. *J. Am. Chem. Soc.* **138**, 5731–5744 (2016).

51. X. Liu, D. Lyu, C. Merlet, M. J. A. Leesmith, X. Hua, Z. Xu, C. P. Grey, A. C. Forse, Structural disorder determines capacitance in nanoporous carbons. *Science* **384**, 321–325 (2024).

52. D. L. McCaffrey, S. C. Nguyen, S. J. Cox, H. Weller, A. P. Alivisatos, P. L. Geissler, R. J. Saykally, Mechanism of ion adsorption to aqueous interfaces: Graphene/water vs. air/water. *Proc. Natl. Acad. Sci. U.S.A.* **114**, 13369–13373 (2017).

53. X. R. Advincula, K. D. Fong, Y. Wang, C. Schran, M. Bonn, A. Michaelides, Y. Litman, Breaking the air-water paradigm: Ion behavior at hydrophobic solid-water interfaces. *J. Am. Chem. Soc.* **148**, 12753–12763 (2026).

54. Y. Wang, H. Luo, X. R. Advincula, Z. Zhao, A. Esfandiar, D. Wu, K. D. Fong, L. Gao, A. S. Hazrah, T. Taniguchi, C. Schran, Y. Nagata, L. Bocquet, M. L. Bocquet, Y. Jiang, A. Michaelides, M. Bonn, Spontaneous surface charging and janus nature of the hexagonal boron nitride-water interface. *J. Am. Chem. Soc.* **147**, 30107–30116 (2025).

55. S. Jiao, C. Duan, Z. Xu, Structures and thermodynamics of water encapsulated by graphene. *Sci. Rep.* **7**, 2646 (2017).

56. Y. Wang, F. Tang, X. Yu, K. Y. Chiang, C. C. Yu, T. Ohto, Y. Chen, Y. Nagata, M. Bonn, Interfaces govern the structure of angstrom-scale confined water solutions. *Nat. Commun.* **16**, 7288 (2025).

57. X. R. Advincula, C. Schran, A. Michaelides, When is nanoconfined water different from interfacial water? *Faraday Discuss*, https://doi.org/10.1039/D5FD00165J (2026).

58. S. Ruiz-Barragan, H. Forbert, D. Marx, Anisotropic pressure effects on nanoconfined water within narrow graphene slit pores. *Phys. Chem. Chem. Phys.* **25**, 28119–28129 (2023).

59. F. Leoni, C. Calero, G. Franzese, Nanoconfined fluids: Uniqueness of water compared to other liquids. *ACS Nano* **15**, 19864–19876 (2021).

60. D. Muñoz-Santiburcio, C. Wittekindt, D. Marx, Nanoconfinement effects on hydrated excess protons in layered materials. *Nat. Commun.* **4**, 2349 (2013).

61. D. Muñoz-Santiburcio, D. Marx, On the complex structural diffusion of proton holes in nanoconfined alkaline solutions within slit pores. *Nat. Commun.* **7**, 12625 (2016).

62. C. Zhang, Z. Yu, R. Car, A. Selloni, Tuning water dissociation at oxide-electrolyte interfaces with electric fields. *Proc. Natl. Acad. Sci. U.S.A.* **122**, e2505929122 (2025).

63. I. Batatia, D. P. Kovacs, G. N. C. Simm, C. Ortner, G. Csanyi, “MACE: Higher Order Equivariant Message Passing Neural Networks for Fast and Accurate Force Fields,” *Advances in Neural Information Processing Systems* (NeurIPS, 2022); https://openreview.net/forum?id=YPpSngE-ZU.

64. X. R. Advincula, K. D. Fong, A. Michaelides, C. Schran, Protons accumulate at the graphene-water interface. *ACS Nano* **19**, 17728–17737 (2025).

65. F. L. Thiemann, C. Schran, P. Rowe, E. A. Müller, A. Michaelides, Water flow in single-wall nanotubes: Oxygen makes it slip, hydrogen makes it stick. *ACS Nano* **16**, 10775–10782 (2022).

66. T. D. Kühne, M. Iannuzzi, M. del Ben, V. V. Rybkin, P. Seewald, F. Stein, T. Laino, R. Z. Khaliullin, O. Schütt, F. Schiffmann, D. Golze, J. Wilhelm, S. Chulkov, M. H. Bani-Hashemian, V. Weber, U. Borštnik, M. Taillefumier, A. S. Jakobovits, A. Lazzaro, H. Pabst, T. Müller, R. Schade, M. Guidon, S. Andermatt, N. Holmberg, G. K. Schenter, A. Hehn, A. Bussy, F. Belleflamme, G. Tabacchi, A. Glöß, M. Lass, I. Bethune, C. J. Mundy, C. Plessl, M. Watkins, J. VandeVondele, M. Krack, J. Hutter, CP2K: An electronic structure and molecular dynamics software package—Quickstep: Efficient and accurate electronic structure calculations. *J. Chem. Phys.* **152**, 194103 (2020).

67. J. P. Perdew, K. Burke, M. Ernzerhof, Generalized gradient approximation made simple. *Phys. Rev. Lett.* **77**, 3865–3868 (1996).

68. S. Grimme, J. Antony, S. Ehrlich, H. Krieg, A consistent and accurate ab initio parametrization of density functional dispersion correction (DFT-D) for the 94 elements H-Pu. *J. Chem. Phys.* **132**, 154104 (2010).

69. M. J. Gillan, D. Alfè, A. Michaelides, Perspective: How good is DFT for water? *J. Chem. Phys.* **144**, 130901 (2016).

70. T. Morawietz, A. Singraber, C. Dellago, J. Behler, How van der Waals interactions determine the unique properties of water. *Proc. Natl. Acad. Sci. U.S.A.* **113**, 8368–8373 (2016).

71. O. Marsalek, T. E. Markland, Quantum dynamics and spectroscopy of Ab initio liquid water: The interplay of nuclear and electronic quantum effects. *J. Phys. Chem. Lett.* **8**, 1545–1551 (2017).

72. A. O. Atsango, T. Morawietz, O. Marsalek, T. E. Markland, Developing machine-learned potentials to simultaneously capture the dynamics of excess protons and hydroxide ions in classical and path integral simulations. *J. Chem. Phys.* **159**, 074101 (2023).

73. J. G. Brandenburg, A. Zen, D. Alfè, A. Michaelides, Interaction between water and carbon nanostructures: How good are current density functional approximations? *J. Chem. Phys.* **151**, 164702 (2019).

74. S. Goedecker, M. Teter, J. Hutter, Separable dual-space Gaussian pseudopotentials. *Phys. Rev. B Condens. Matter* **54**, 1703–1710 (1996).

75. A. H. Larsen, J. J. Mortensen, J. Blomqvist, I. E. Castelli, R. Christensen, M. Dułak, J. Friis, M. N. Groves, B. Hammer, C. Hargus, E. D. Hermes, P. C. Jennings, P. B. Jensen, J. Kermode, J. R. Kitchin, E. L. Kolsbjerg, J. Kubal, K. Kaasbjerg, S. Lysgaard, J. B. Maronsson, T. Maxson, T. Olsen, L. Pastewka, A. Peterson, C. Rostgaard, J. Schiøtz, O. Schütt, M. Strange, K. S. Thygesen, T. Vegge, L. Vilhelmsen, M. Walter, Z. Zeng, K. W. Jacobsen, The atomic simulation environment—A Python library for working with atoms. *J. Phys. Condens. Matter* **29**, 273002 (2017).

76. PLUMED consortium, Promoting transparency and reproducibility in enhanced molecular simulations. *Nat. Methods* **16**, 670–673 (2019).

77. J. Kästner, W. Thiel, Bridging the gap between thermodynamic integration and umbrella sampling provides a novel analysis method: "Umbrella integration". *J. Chem. Phys.* **123**, 144104 (2005).

78. D. P. Kovács, J. H. Moore, N. J. Browning, I. Batatia, J. T. Horton, Y. Pu, V. Kapil, W. C. Witt, I. B. Magdău, D. J. Cole, G. Csányi, MACE-OFF: Short-range transferable machine learning force fields for organic molecules. *J. Am. Chem. Soc.* **147**, 17598–17611 (2025).

79. W. C. Witt, Symmetrix (2025); https://github.com/wcwitt/symmetrix.

80. A. P. Thompson, H. M. Aktulga, R. Berger, D. S. Bolintineanu, W. M. Brown, P. S. Crozier, P. J. in ‘t Veld, A. Kohlmeyer, S. G. Moore, T. D. Nguyen, R. Shan, M. J. Stevens, J. Tranchida, C. Trott, S. J. Plimpton, LAMMPS—A flexible simulation tool for particle-based materials modeling at the atomic, meso, and continuum scales. *Comput. Phys. Commun.* **271**, 108171 (2022).

81. X. R. Advincula, Y. Litman, K. D. Fong, W. C. Witt, C. Schran, A. Michaelides, Research data supporting: “How reactive is water at the nanoscale and how to control it?” (2026); https://doi.org/10.17863/CAM.125023.

82. A. H. Castro Neto, F. Guinea, N. M. R. Peres, K. S. Novoselov, A. K. Geim, The electronic properties of graphene. *Rev. Mod. Phys.* **81**, 109–162 (2009).

83. K. D. Fong, B. Sumić, N. O’Neill, C. Schran, C. P. Grey, A. Michaelides, The interplay of solvation and polarization effects on ion pairing in nanoconfined electrolytes. *Nano Lett.* **24**, 5024–5030 (2024).

84. Y. Litman, V. Kapil, Y. M. Y. Feldman, D. Tisi, T. Begušić, K. Fidanyan, G. Fraux, J. Higer, M. Kellner, T. E. Li, E. S. Pós, E. Stocco, G. Trenins, B. Hirshberg, M. Rossi, M. Ceriotti, i-PI 3.0: A flexible and efficient framework for advanced atomistic simulations. *J. Chem. Phys.* **161**, 062504 (2024).

85. G. Bussi, D. Donadio, M. Parrinello, Canonical sampling through velocity rescaling. *J. Chem. Phys.* **126**, 014101 (2007).

86. J. VandeVondele, J. Hutter, Gaussian basis sets for accurate calculations on molecular systems in gas and condensed phases. *J. Chem. Phys.* **127**, 114105 (2007).

87. Y. Wei, B. Wang, J. Wu, R. Yang, M. L. Dunn, Bending rigidity and gaussian bending stiffness of single-layered graphene. *Nano Lett.* **13**, 26–30 (2013).

88. J. Kästner, W. Thiel, Analysis of the statistical error in umbrella sampling simulations by umbrella integration. *J. Chem. Phys.* **124**, 234106 (2006).

89. N. Kastelowitz, J. C. Johnston, V. Molinero, The anomalously high melting temperature of bilayer ice. *J. Chem. Phys.* **132**, 124511 (2010).

90. J. Zubeltzu, F. Corsetti, M. V. Fernández-Serra, E. Artacho, Continuous melting through a hexatic phase in confined bilayer water. *Phys. Rev. E* **93**, 062137 (2016).

91. T. Dufils, C. Schran, J. Chen, A. K. Geim, L. Fumagalli, A. Michaelides, Origin of dielectric polarization suppression in confined water from first principles. *Chem. Sci.* **15**, 516–527 (2024).

92. B. Das, S. Ruiz-Barragan, D. Marx, Deciphering the properties of nanoconfined aqueous solutions by vibrational sum frequency generation spectroscopy. *J. Phys. Chem. Lett.* **14**, 1208–1213 (2023).

93. S. Ruiz-Barragan, D. Muñoz-Santiburcio, S. Körning, D. Marx, Quantifying anisotropic dielectric response properties of nanoconfined water within graphene slit pores. *Phys. Chem. Chem. Phys.* **22**, 10833–10837 (2020).

94. S. Ruiz-Barragan, D. Muñoz-Santiburcio, D. Marx, Nanoconfined water within graphene slit pores adopts distinct confinement-dependent regimes. *J. Phys. Chem. Lett.* **10**, 329–334 (2019).

95. H. Duan, Z. Ying, L. Tian, Y. Cheng, L. Shi, Aqueous proton transportation in graphene-based nanochannels. *Langmuir* **38**, 15413–15421 (2022).

96. S. J. Stuart, A. B. Tutein, J. A. Harrison, A reactive potential for hydrocarbons with intermolecular interactions. *J. Chem. Phys.* **112**, 6472–6486 (2000).

97. M. Ceriotti, W. Fang, P. G. Kusalik, R. H. McKenzie, A. Michaelides, M. A. Morales, T. E. Markland, Nuclear quantum effects in water and aqueous systems: experiment, theory, and current challenges. *Chem. Rev.* **116**, 7529–7550 (2016).

98. D. W. Lim, X. R. Advincula, W. C. Witt, A. Michaelides, F. L. Thiemann, C. Schran, Strain-dependent wetting of graphene. arXiv:2601.20134 [cond-mat.mes-hall] (2026).
